# Preliminary Evidence of Sleep Improvements Following Psilocybin Administration, and their Involvement in Antidepressant Therapeutic Action

**DOI:** 10.1007/s11920-024-01539-8

**Published:** 2024-11-13

**Authors:** Matthew J. Reid, Hannes Kettner, Tessa F. Blanken, Brandon Weiss, Robin Carhart-Harris

**Affiliations:** 1 Behavioral Sleep Medicine Research Laboratory, Department of Psychiatry and Behavioral Sciences, Johns Hopkins School of Medicine, 5510 Nathan Shock Drive, Suite 100, Baltimore, MD 21224 USA; 2 Johns Hopkins Precision Medicine Center of Excellence (PMCoE) for Depression, Johns Hopkins Hospital, Baltimore, MD USA; 3 Department of Neurology, University of California San Francisco, San Francisco, CA USA; 4 Department of Psychological Methods and Clinical Psychology, University of Amsterdam, Amsterdam, Netherlands; 5 Center for Psychedelics and Consciousness Research, Department of Psychiatry and Behavioral Sciences, Johns Hopkins School of Medicine, Baltimore, MD USA; 6 Department of Medicine, Centre for Psychedelic Research, Imperial College London, London, UK

**Keywords:** Sleep, Psilocybin-assisted therapy, ADOPT, Depression, Mood Disorders, Psilocybin, Psychedelic Medicine

## Abstract

**Purpose of the study:**

Psilocybin is a rapidly-emerging treatment for depression, yet its impact on sleep is not well understood. We sought to explore the literature on sleep and psilocybin use, and explore the topic using our own primary data.

**Findings:**

Whilst clinical trials demonstrate large depressive symptom improvements, the impact of psilocybin on sleep quality or insomnia symptoms, has not been directly studied. Using our own preliminary-data we demonstrated that both depressive-symptoms and sleep-disturbances decreased significantly following psilocybin use, though sleep improvements were smaller compared to depressive symptoms. More severe sleep-disturbances at baseline were linked to lower probability of depression remission, underscoring a potential interaction between sleep and psilocybin’s efficacy.

**Summary:**

Addressing sleep disturbances could enhance therapeutic outcomes in psilocybin-assisted therapy and could lead to more effective, personalized treatment-strategies. Future research should focus on populations with sleep disorders, and on examining causal-pathways of sleep physiology’s impact on psilocybin efficacy.

**Supplementary Information:**

The online version contains supplementary material available at 10.1007/s11920-024-01539-8.

## Introduction

Psilocybin has rapidly emerged as the leading therapeutic candidate amongst the new wave of psychedelic medicine, particularly within the context of major depressive disorder [1, 2]. Despite its pre-regulatory status, the drug is widely accessible in organic-form due to the recent legislative initiatives of several countries and US-states, giving rise to self-medication practices and the emergence of retreat-style guided psilocybin experiences [3] to fill this need. Although the majority have been marketed towards personal-growth and wellness, one of the most frequently cited motivations for undertaking self-guided or retreat-style guided psilocybin experiences is self-medication for pre-existing depressive symptoms, amongst other physical and mental complaints [4–6]. Despite their flourishing popularity amongst those seeking alternative routes to improved depressive symptoms [7] very few empirical studies have been conducted amongst individuals participating in retreat-style psychedelic experiences.

Notwithstanding the magnitude and longevity of depressive symptom improvements observed in psychedelic clinical trials, up to 30% of participants in research studies fail to demonstrate adequate response [2], suggesting that person-level or environmental factors may determine likelihood of deriving a clinically-meaningful benefit. This may be explained, in part, by the heterogenous nature of depressive symptoms, which span several interconnected yet highly divergent constructs, such as mood, appetite, cognition, and sleep. Perhaps the most scarcely described of these factors amongst psychedelic research is sleep, and currently very little is known about whether psilocybin alters sleep, particularly beyond acute states of drug metabolism window. Only one small study [8] has examined the effects of non-therapeutic psilocybin administration on sleep-EEG measures amongst 19 healthy humans, reporting prolonged REM latency following a single daytime administration session. The same study also reported a reduction in delta/slow-wave power during SWS in the first sleep cycle, suggesting alterations in homeostatic sleep drive. However, these sleep-EEG recordings were observed the night immediately after psilocybin administration, and therefore the long-term effects of psilocybin on sleep remain entirely unknown. This is pertinent, as the active metabolites and transient alterations in receptor trafficking [9] which are present during the acute psilocybin administration period are likely to influence sleep in ways that are different to any neuroplastic changes which may be present several weeks after the drug has been metabolized. Similar findings of prolonged REM latency have emerged from animal models [10], following administration of psilocin (the pharmacologically active metabolite of psilocybin) in mice.

However, in animal studies enhanced oscillations in a narrow window of the delta (0.5-4 Hz) frequency band, around 4 Hz were observed, which contrasts with the reduction in 0.5-4 Hz power observed in humans[8]. To our knowledge, no human-subjects study has investigated whether improvements in self-report sleep quality are observed following Administration Of Psilocybin-based Therapeutics (ADOPT), or whether these changes are sustained beyond the period of acute administration. Using naturalistic survey data obtained from participants who took part in facilitated psilocybin sessions within retreat settings, or guided psilocybin experiences, we aimed to examine the impact of psilocybin on sleep and other depressive symptoms at 2-weeks and 4-weeks post-intervention, and the possible impact of baseline sleep quality on psilocybin-mediated antidepressant response. Indeed, given sleep’s impact on many facets of emotional wellbeing, including mood and anxiety [11], poor sleep quality has considerable potential to contribute to the physiological ‘set’ of an individual’s psychedelic experiences, which may contribute to their overall long-term benefits. We explored two key questions; 1) Does guided psilocybin administration lead to sustained improvements [up to 1 month] in sleep and depressive symptoms. 2) Do baseline sleep disturbances, and or changes in sleep symptoms predict improvement in depressive symptoms following psilocybin administration.

### Experimental Procedures

Data were obtained from participants (*n* = 886) who identified an intent to participate in psychedelic use in the near future (for detailed methods see [12]). Participants were required to be 18 years or older, have sufficient English comprehension, and must have indicated that they were about to participate in a retreat, ceremony or other guided experience involving the use of a classic psychedelics containing 5-HT2A receptor agonist (e.g., Psilocybin, DMT, Mescaline, or LSD). Data were collected prospectively from the participants using online surveys completed before, during, and for up to four weeks after their psychedelic experience. We restricted our analyses to respondents who reported psilocybin use, given that it has the greatest clinical relevance to depression (*n* = 653). Ethical approval was granted by the joint research compliance office and the Imperial College Research Ethics Committee (ICREC reference 181C4346). Data originated from 111 centers, some of which contributed substantially to the proportion of the sample (max = 297 participants), whereas others provided only a single or several participants.

#### Assessment of Depressive and Sleep Symptoms

We measured depressive symptom severity using the Quick Inventory for Depressive Symptoms (QIDS [13]), a well-validated 16 item scale which covers the key diagnostic domains of depressive symptomology. The QIDS includes a sleep sub-scale, comprised of four items assessing sleep onset insomnia (Item 1 ‘*Difficulty falling asleep’*), sleep maintenance insomnia (Item 2 ‘*Sleep during the night’*), early morning insomnia (Item 3 ‘*Waking up too early’*), and hypersomnia (Item 4 *‘Sleeping too much’*). In keeping with standard scoring instructions for the QIDS, we used the maximum value of the individual item scores (each scored 0 [no disturbance] to 3 [max disturbance]) of these four items to define the severity of sleep disturbance symptoms (max score 27). Although the QIDS was designed for the assessment of global symptom severity, previous studies have used the QIDS-sleep sub-scale in isolation to quantify sleep symptom improvements in other rapid acting antidepressants, as well as the relationship between depression related sleep-disturbance and functional outcomes [14–17]. We used the QIDS total score to measure depressive symptom severity (QIDS-*depression*). Naturally, as the score from the sleep sub-scale contributes to the total QIDS total score, a significant relationship between QIDS-*sleep* and QIDS-*total* is implicit. Therefore, we excluded the sleep scale from the total QIDS score (i.e., QIDS-*depression*_=_ QIDS-*total* – QIDS-*sleep*). We defined depressive symptom remission at both two-week and four-weeks as a QIDS-*depression* reduction > 50% relative to baseline, and QIDS-*total* score < 6 at the respective timepoint.

### Statistical Analyses

Sleep and depressive symptoms share a highly complex and bi-directional relationship. Therefore, to better aid in parsing these pathways, we used a nested sequence of a priori and confirmatory post hoc models that we further outline below. We first examined univariate change in sleep and depressive symptoms using random-effects models. Next, we used structural equation models to examine contemporaneous and longitudinal interactions between sleep and depressive symptoms at baseline, two-weeks and four-weeks. Finally, we probed symptom-level effects and predictors of treatment response using a novel network analysis approach, adapted previously for use in sleep and mental-health symptom networks [18].

#### Mixed-Effects Models

Outcomes were change in QIDS-*depression* total score and QIDS-*sleep* total score at two weeks and four weeks. Fixed effects in both models included timepoint (baseline, two-weeks, four-weeks), and the respective outcome measure at baseline to account for potential confounding of initial symptom severity. We performed model diagnostics using visual assessments of the normality and heteroskedasticity of residuals (see supplement for further information on diagnostics). We interpreted unadjusted models as our primary outcome, to optimise model parsimony and facilitate hypothesis generation, and to aid in comparison with future studies, given this early exploratory stage. However, we conducted pre-planned sensitivity analyses to conduct a secondary corrected model by incrementally adjusting for available covariates (Age, ethnicity, marital status, employment, income, country of retreat, number of sessions, psilocybin dose, retreat-center) exploring their impact on model fit and interpretation, which we report in full in the supplement. To limit overfitting and ensure appropriate variance component estimates, we based our model selection on both the Restricted Maximum Likelihood (REML) and Bayesian Information Criterion (BIC) values. REML was used to provide unbiased estimates of variance components, while BIC helped identify the most parsimonious model by balancing fit and complexity.

Given that large variations in sample size can give rise to spurious fixed effects in nested random-effects models, we also performed a Bayesian-simulation using Montecarlo Markov Chains to generate robust estimates of expected variation in our two outcome variables (sleep and depressive symptoms) across the range of center sample sizes, to distinguish any meaningful center-based effects from natural variance. Bootstrapping was performed on the uncorrected, and best-fitting corrected models to obtain estimates of the stability of the 95% Bootstrapped Confidence Intervals (BCI) of the fixed-effect coefficients. Missing outcome data at each two weeks (53%) and four-week (55%) were determined to be Missing Not at Random (MNAR), as greater observed values for: age, number of sessions, duration of retreat, and outcomes at baseline were significantly associated with increased missingness. Therefore, we performed imputation sensitivity analyses using Baseline Observation Carried Forward (BOCF) to simulate how a ‘worse-case’ scenario might bias our findings, in the event that that those with missing values demonstrated no improvement. We also used a deep-learning imputation approach to impute values based on auxiliary variables predictive of outcome (see supplement for methods).

#### Structural Equation Models

Structural equation modeling was used to examine the dynamic relationships between QIDS-depression (represented as *qids* in formulae notation – without sleep items) and sleep quality [QIDS-sleep] across three time points. A cross-lagged panel framework was used to model the variance and covariance (Σ) where *β*_*ij*_​ and *γ*_*ij*_​ represent the coefficients for the QIDS-depression and QIDS-sleep variables, capturing the lagged events across different time points. Error terms *ε-qids*​ and *ε-sleep*​ account for unobserved factors influencing depressive symptoms (*qids [without sleep]*) and sleep quality (*sleep*) at each time point. This approach allowed us to examine how each variable influences the other across time and to probe both the temporal order effects and potentially causal dynamics between *sleep* and *qids* variables measured at multiple time points. Path coefficients were estimated using Maximum Likelihood Estimation and reported as standardized λ values. Lagged analyses controlled for the baseline value of the lagged variable (i.e., Sleep_0_), as well as the value of the covariate (i.e., Depression_2_) at the same time point (i.e., Depression^3^ ~ Depression^2^ + Sleep^0^ + Sleep^2^). Diagnostics and methods are reported in the supplement.

#### Symptom Network Analysis

To investigate symptom-specific relationships between individual QIDS items and overall depressive-symptom improvement, we adapted a Network Interventional Analysis [18] and estimated, a Mixed Graphical Model [19] at baseline where each node is represented by one of the 16 QIDS items. We then estimated their conditional relationship with remission likelihood by introducing a remission node, which represented whether or not participants experienced a remission at two weeks (network 1) or four weeks (network 2).

Network analyses were used to generate mixed graphical models [20] via K-degree nodewise regression, which estimates the conditional dependencies between all nodes in the network. LASSO regularization was used to calculate the adjusted edge weights of nodewise regressions. In accordance with previous recommendations [21, 22]. We generated 1000 new resampled networks using the ‘resample’ function of the MGM package, and verified the accuracy and stability of the estimated network models [23] by assessing the probability (%) that a given edge (i.e., link between symptoms) was present in the network after resampling, as well as the strength of the included links, by assessing the range of the adjusted edge weights observed during resampling. To facilitate comparisons between edges, we performed post-hoc edge difference tests ( Bootnet::differenceTest() in R), to compare the magnitude of differences between observed edges.

In our network analyses, all variables (e.g., the QIDS items and the response variables [*remission*]) are included as individual *nodes* and visualised in a network. *Nodes* are linked by *edges* that represent conditional-dependence among them, *i.e*., the unique association between two variables after conditioning on all other variables in the network.. Therefore, the relationships between the individual symptoms and remission we report implicitly controls for the confounds of the remaining symptoms. We used the magnitude and path of adjusted edge weights to interpret the magnitude of these relationships, whereby positive (*Greater likelihood of remission*) and negative (*Smaller likelihood of remission*) relationships were represented by blue (positive) and red (negative) edges.

In order to quantify remission cases, we confined the sample used for the network analysis to those who demonstrated clinically significant depressive symptoms at baseline (*n* = 392), as defined as a score > 5 on the QIDS total score, a common cutoff for ‘mild depressive symptoms’[13]. For transparency, we also report networks derived from the whole sample in the supplement. N.B: The multilevel models and SEMs were first performed using the entire sample, followed by a sub-group analysis with clinically significant symptoms. We further probed the extent to which any relationships between sleep symptoms and depressive symptom improvement may have been influenced by the presence of sleep items in the outcome variable, after reintroduction of sleep item. We conducted primary analyses uncorrected but performed sensitivity analyses by sequentially introducing additional nodes for study center, psilocybin dose, and the number of sessions to examine their impact on the network relationships demonstrated in our primary analyses (see supplement).

## Results

**Fig. 1:**
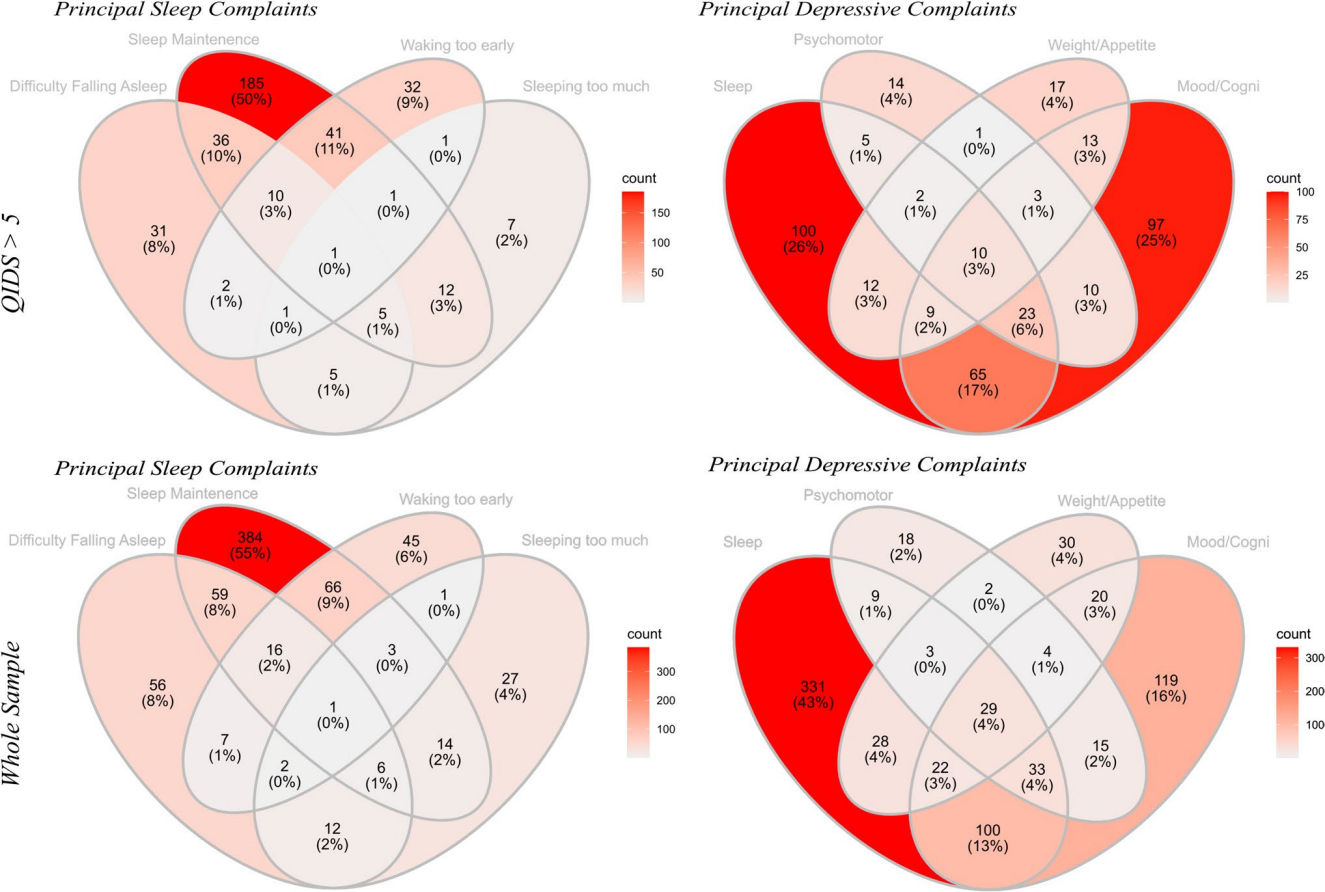
Frequency of symptom-reports at baseline. Top row = participants with total QIDS-16 scores deemed within the clinically significant range (> 5) at baseline. Bottom Row = All participants. Principal Sleep Complaints: diagrams represent the frequency of the most severe sleep related symptom(s) in participants who report at least one sleep symptom (sleep score > 0). Overlapping regions indicate equal severity. Principle depressive complaints: Diagrams represent the highest scoring QIDS subscale for participants who reported at least one (i.e., QIDS-total > 0) depressive symptom. Values were normalized according to maximum possible score for each sub-scale. Overlapping regions indicate that both symptoms were equal in severity. Sleep = items 1,2,3,4, Psychomotor = items 15, 16 & 14, Weight/appetite = items 6,7,8,9, Mood/Cogni = items 5,6,10,11. Overlapping regions indicate equal severity

653 participants provided complete QIDS responses, prior to administration with psilocybin, of whom 370 had complete QIDS data at the two-week assessment and 354 at four week follow up. Demographic information collected during the baseline survey is presented in supplementary Table 1. Briefly, participants were primarily white (90.7%), male (55.6%), university educated (84.2%) and employed (78.1%). The overall mean QIDS-total scores (6.78, ^*SD*^4.67) for the sample at baseline was indicative of mild-moderate depressive symptoms with 392 (60%) participants exceeding the QIDS diagnostic cutoff (QIDS-total > 5) for clinically significant depressive symptoms. 97 (14.8%) participants reported having received a previous diagnosis of MDD, and 19 (2.9%) reported having previously been diagnosed with a bipolar spectrum disorder (see supplementary Table 2 for full list of diagnoses). Sleep complaints were prevalent amongst participants, with all 653 participants reporting at least some degree (score ≥ 1 out of 3) of sleep disturbance at baseline (mean = 2.03, ^*SD*^0.71). Remarkably, amongst these participants, sleep disturbance was the principal depressive symptom (26%), marginally outnumbering mood/cognition complaints (25%). Of the participants who reported at least one sleep complaint (score ≥ 1), the symptom *Sleep onset insomnia*^*Qids-1*^ was the principal complaint in 43% of cases, followed by *hypersomnia*^*Qids-4*^ (16%), *early morning awakenings*^*Qids-3*^ (30%) and *sleep maintenance insomnia*^*Qids-2*^ (18%) (Fig. 1).

### Does Psilocybin Improve Sleep Disturbance as Well as Overall Depressive Symptoms?

#### Sleep

Our uncorrected models demonstrated a significant decrease in sleep disturbances (possible range 0–3) from baseline (*mean* = 2.03, [^*SD*^0.71]), to two weeks (*β* =-0.09 [^*SE*^0.04] 95% CI: -0.16 to -0.02, *p* = 0.01, | *mean* = 1.67, [^*SD*^0.86]), and four weeks (*β* =-0.13 [^*SE*^0.05] 95% CI: -0.17 to -0.02, *p* = < 0.001 **|** *mean* = 1.67 [^*SD*^0.75]), relative to baseline. The magnitude of the decrease in sleep disturbance was small (Cohen’s *d:* Two Weeks =-0.13 | Four Weeks = -0.18) but larger effects were observed when the analysis was restricted to participants who demonstrated moderate to severe (sleep scale ≥ 2) sleep disturbances (*p* < 0.001, Cohen’s *d* =-0.39) at baseline. Effects were also sustained following sensitivity analyses, remaining significant after 1): being performed on the dataset imputed using multiple imputations via deep learning (Two weeks *d* = -0.18*, p* < 0.001 | Four weeks *d* = -0.14, *p* < 0.001) and also ‘worst-case’ imputation using BOCF at two weeks (*p* = 0.024, *d* = 0.08) but not four weeks (*p* = 0.113, *d* = -0.05) albeit with small effect sizes. Incremental adjustments concluded that there was significant effect of the retreat center on the response variable. Nevertheless, including covariates in the model did not improve fit indices, or substantively alter fixed effect estimates (see supplement). Accordingly, our funnel-plot simulations demonstrated that the majority of the observed variance between centers could be explained by natural sampling variability due to differences in center size (supplementary Fig. 2).

#### Depressive Symptoms (QIDS-depression)

Our uncorrected models demonstrated a significant decrease in depressive symptoms (possible range: 0–27) from baseline (mean = 6.78, [^*SD*^4.67]) at both two weeks (*β* = -2.24 [^*SE*^0.15] 95% CI: -2.52 to -1.93, mean QIDS = 4.27, [^*SD*^1.72]) and four weeks (*β* = -3.50 [^*SE*^0.15] 95% CI: -3.79 to -3.19, mean QIDS = 3.04, [^*SD*^3.05]). The magnitude of the decrease in depressive symptoms was moderate to large (Cohen’s *d* =-0.39, 4 weeks = -0.61). Larger effect sizes were observed at two weeks (*β* = -5.95 [^*SE*^0.24] 95% CI: -6.43 to -5.48) and four weeks (*β* =-7.90 [^*SE*^ 0.22] 95% CI: -8.37 to -7.40) when restricting analyses to participants with clinically significant (> 5) QIDS-total scores at baseline (Cohen’s *d* = -1.20 & -1.54). Complete model parameters are reported in Supplementary Table 3. This effect was sustained following sensitivity analyses, remaining significant after: 1) re-inclusion of the sleep items to the QIDS-total score (Two weeks: *d* = -0.45, < 0.001 | Four weeks *d* = -0.16, *p* = < 0.001), and was also significant when performed on datasets imputed by ‘best-case-imputation’ (MICE) (Two Weeks: *p* < 0.001, *d* = -0.37 | Four Weeks: *p* < 0.001, *d* = -0.56) and also ‘worst-case-imputation’ methods (BOCF) (Two Weeks = *p* < 0.001, *d* = -0.52 | Four weeks *p* < 0.001, d = -0.34). Incremental adjustments concluded that none of the demographic covariates were significantly associated with the outcome (supplementary Table 4). Furthermore, in keeping with findings obseved for QIDS-sleep, inclusion of these covariates did not improve fit based on BIC and REML values, relative to unadjusted models. Moreover, our funnel simulation models demonstrated that the majority of the observed variance between centers could be explained by natural sampling variability arising from differences in center size (supplementary Fig. 2), as opposed to any substantive differences in outcome between centers.

### Do Those with Worse Sleep Disturbance Experience Poorer Depressive Symptom Outcome?

**Fig. 2:**
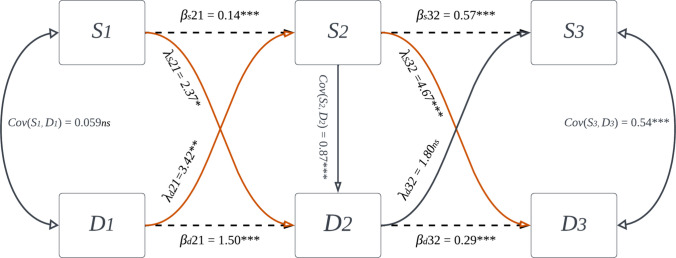
Cross-lagged panel model of temporal dependencies between sleep and depression. Boxes = observed measurements. Dashed paths = autoregressive variance. Red and black sigmoidal paths = cross-lagged paths. S = sleep, D = depressive symptoms, 1,2,3 = relative timepoints. 1 = baseline, 2 = two weeks, 3 = four weeks. Red paths depict a significant negative/inverse relationship between lagged variables. *** = *p* < 0.001, ns = not statistically significant (*p* > 0.05). Error-terms and covariates omitted for visualization

Our cross-lagged paths ($\lambda$) demonstrated that observed sleep values, controlling for observed depression values at the same timepoint (*i*) and at baseline (*i*_0_), predicted lagged depression values at the subsequent timepoint (two weeks: $\lambda =2.37,$ *β* = 0.27, *p* = 0.018 | Four weeks: $\lambda =4.67,$ *β* = 0.30, *p* < 0.001), whereas depressive symptoms at two weeks did not predict lagged sleep symptoms at four weeks ( $\lambda =1.80,$ *β* = 0.02, *p* = 0.071) and baseline depressive symptoms only predicted lagged sleep symptoms at two weeks ($\lambda =3.42,$ *β* = 0.07, *p* = 0.001, see Fig. 2). After demonstrating temporal relationships between sleep and depressive symptoms across the timepoints, we estimated network models to investigate the relationship between individual sleep items and depressive symptom remission at two weeks and four weeks.

#### Worse Sleep at Baseline is Associated with Reduced Depressive Symptom Remission Post-Intervention (2 weeks)

**Fig. 3:**
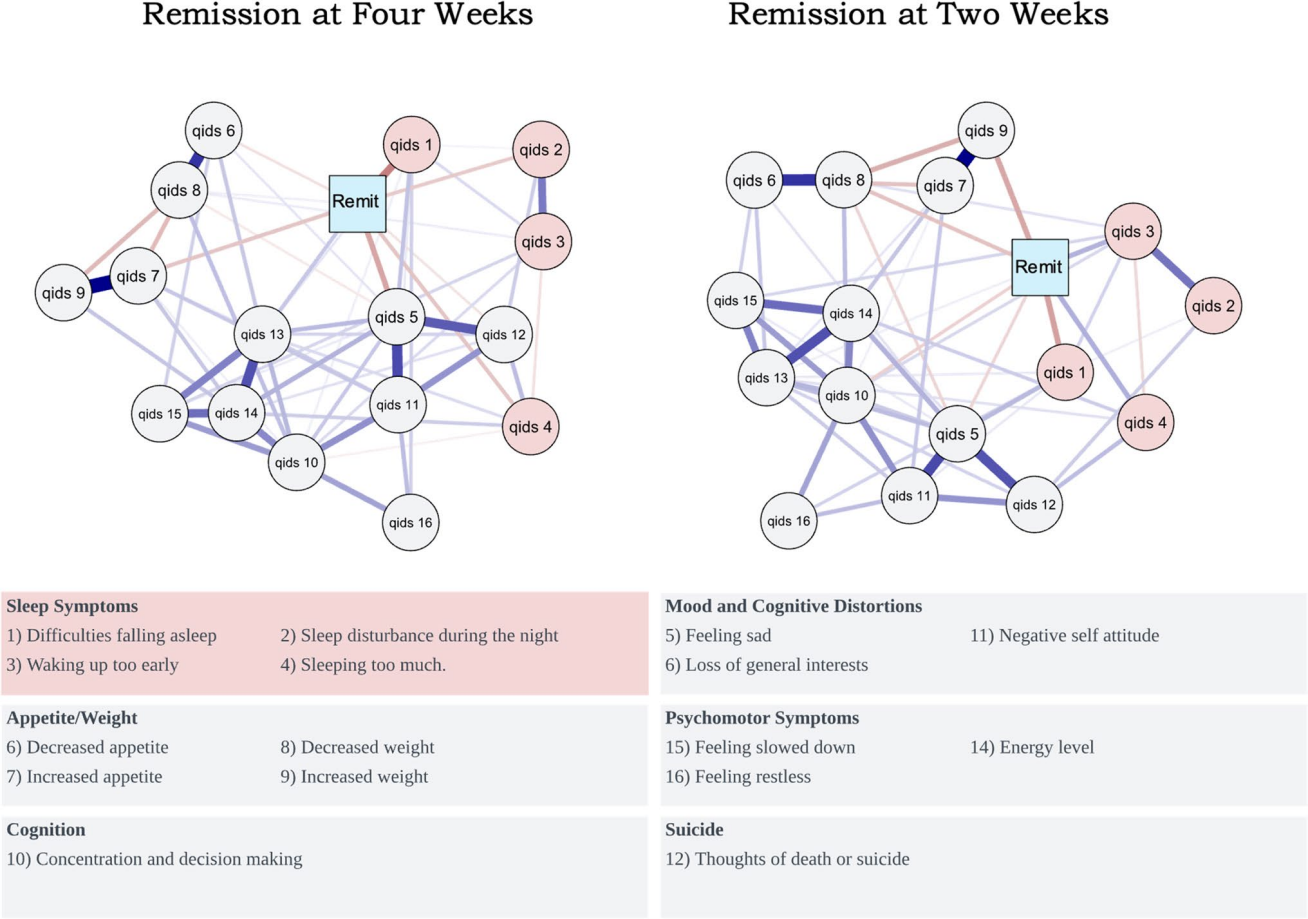
Symptom Networks: Mixed Graphical Models of Symptom Networks generated by K-degree nodewise regression. In network analyses, a direct link (i.e., an ‘Edge’) between nodes (circles) and the lagged outcome node [‘Remit’/Square] represents a direct relationship between the two variables, after controlling for relationships amongst all other nodes. The thickness and opacity of the edge connecting nodes represents the magnitude of the relationship (thicker = stronger), after accounting for other nodes. Red edges indicated a negative/inverse association between the symptom and remission (i.e., reduced likelihood of remission), whereas blue edges indicate a positive (i.e., increased likelihood of remission) association. Remit = Remission of depressive symptoms at timepoint (QIDS < 6). Networks were cross-validated using k-folds trained on 10 datasets. Table of Network weights is presented in supplement. At two weeks, all of the QIDS -Sleep items, except QIDS -2 (Sleep Maintenance Insomnia) were linked to likelihood of depression. At four weeks, the symptom [of all 16] most strongly linked to remission was Qids-1 – Sleep Onset Latency, despite diminishing links between the other sleep items and remission

Figure 3 shows a direct link between three sleep-symptoms at baseline and likelihood of depressive symptom remission at two weeks. *Sleep onset insomnia*^*Item-1*^ (network weight = 0.145) was the baseline-symptom (including non-sleep related symptoms) most strongly associated with reduced likelihood of remission at two-weeks. *Hypersomnia*^*Item-4*^ (network weight = 0.122) and *Early morning awakenings*^*Item-3*^ (network weight = 0.119) were moderately associated with an increased likelihood of experiencing remission at two weeks (Fig. 3). After simulating 1000 resampled datasets, bootstrapped parameters indicated high stability of these relationships within the observed networks. *Sleep onset insomnia*^*Item-1*^ remained linked to remission in 100% of resampled networks. *Hypersomnia*^*Item-4*^ (90%), *early morning awakenings*^*Item-3*^ (77%) demonstrated adequate stability (see supplement for remaining symptoms).

#### Worse Sleep at Baseline is Associated with Reduced Depressive Symptom Remission at Follow-up (4 weeks).

At four weeks post-administration, three of the four sleep scale items were directly linked with the likelihood of experiencing a remission in depressive symptoms., whilst also controlling for all other symptoms within the network (Fig. 3); (*Sleep onset insomnia*^*Item-1*^ (network weight = 0.200), *Sleep maintenance insomnia*^*Item-2*^ [network-weight = 0.083], and *Hypersomnia*^*Item-4*^ [network-weight = 0.090]). Simulations demonstrated that these effects were robust to resampling. After 1000 bootstrap resamples, sleep symptoms remained robustly associated with remission within the networks. Sleep onset insomnia and *hypersomnia*^*Item-4*^ remained associated with reduced likelihood of remission in 98% and 96% (respectively) of the simulated networks. *Sleep maintenance insomnia*^*Item-2*^ demonstrated modest stability during simulations and were linked to remission in 50% of resampled networks respectively (see supplement for remaining symptoms). In planned sensitivity analyses, we explored the impact that including information about dose, center location, and number of sessions had on the network edges reported above. Introducing these covariates did not substantively alter the networks, nor the interpretation of relationship between network nodes and remission. We observed similar findings after re-introducing the sleep items which were removed from the total QIDS scores used to calculate remission rates (supplement page 10).

## Discussion

Here we provide preliminary evidence of sleep’s potential role in the therapeutic effects of psilocybin. We demonstrated: 1) Significant improvements in sleep disturbances for up to four weeks after psilocybin use, 2) Residual sleep disturbance following the psilocybin interventions predicted depressive symptoms at subsequent timepoints whereas post-treatment depressive symptoms failed to predict subsequent changes in sleep. 3) Participants presenting with sleep disturbances at baseline were less likely to experience remission of depressive symptoms. All four symptoms of sleep disturbance were associated with reduced likelihood of remission, albeit to varying degrees. Strikingly, at-four weeks, sleep onset insomnia was the symptom (of all 16 QIDS items) most strongly linked to reduced likelihood of remission. Of further note, the strength of these network connections between sleep symptomology and remission strengthened between the two week and four-week timepoints, whilst the remaining edges decayed and links with other symptom items which were present at two weeks disappeared. Taken together, these observations provide convergent evidence of a potentially prominent link between sleep and psilocybin’s therapeutic action.

Conceptually, these findings could be interpreted through three plausible pathways: 1) sleep improvements may be causally involved in the therapeutic pathway of psilocybin’s impact on depressive symptoms, either directly or indirectly, 2) poor sleep may directly interfere with the physiological mechanisms which underly psilocybin’s therapeutic action, 3) sleep disturbances may represent a trait or endophenotype present amongst individuals who are likely to demonstrate poor therapeutic response, regardless of any sleep-related mechanistic action.

### Potential for a Direct Causal Pathway Linking Sleep and Outcome for Psilocybin-Assisted Therapy?

Data from our structural equation models indicated that the paths linking sleep to subsequent improvements in depression were substantially more robust than those linking depression to sleep, suggesting that the sleep improvements observed in the sample did not occur as a downstream consequence of improving depression. This theory is supported by several lines of evidence from the literature which indicate that improving sleep in patients with depressive disorders results in large improvements in depressive symptoms [24–27], and that CBT for insomnia is equally as effective at reducing depressive symptoms as CBT specifically designed to target depression [28]. Furthermore, self-report sleep improvements have previously been demonstrated to mediate improvements in depressive symptoms following treatment with other rapid-acting antidepressants, including IV-ketamine [14]. Given this established causal evidence, it seems reasonable to postulate that a similar effect would be observed with ADOPT. Nevertheless, the question regarding the direct or indirect nature of this pathway remains uncertain. Based on the diversity of its impact on numerous physical and psychological systems, sleep could equally exert therapeutic action through indirect (e.g., improved sleep → reduced fatigue → increased social interaction) or direct pathways (e.g., promoting neuroplasticity or reducing inflammation), or indeed any combination of direct and indirect pathways.

Of note, whilst the significant improvements in sleep we observed were robust, they were modest (*d* = -0.13 to -0.18) when compared with those observed in depressive symptoms (*d* = -0.89), and more importantly, smaller in magnitude than the effects demonstrating that improvements in sleep predicted improvements in depressive symptoms two weeks later (*d* = -0.30). This discrepancy may reflect a highly complex network of direct and indirect pathways with additive impact on depression. Future studies should seek to parse these causal mechanistic pathways by incorporating outcomes across the breadth of clinical (i.e., insomnia symptoms), behavioral (i.e., sleep timing and patterns), and physiological spectra (i.e., sleep-EEG) within randomized controlled trials, and other causal designs.

### Could sleep Disruption be a Therapeutic Blockade for Psilocybin’s Antidepressant Effects?

Curiously, sleep disturbances represented one of the few symptoms specifically associated with diminished, as opposed to enhanced, likelihood of remission. Although other symptoms, including depressed-mood, were also linked to lower likelihood of remission in our networks, sleep-disturbances are arguably more easily modifiable, and hence hold the greatest prospects for targeted intervention. One possible explanation for the relationship between the severity of sleep disturbance and poor depressive symptom improvement may be that chronic sleep disruption interferes with biological or psychological mechanisms responsible for the therapeutic action of psilocybin. Sleep is a major contributor to many of these putative mechanisms including; neuroplasticity, synaptic downscaling and potentiation [29, 30], as well as those involved in overall brain health, such as glymphatic clearance of waste metabolites from the brain [31]. Similarly, sleep disruption is known to increase anxiety states [11], reduce positive affect [11, 32], and impair memory consolidation in both healthy [11] and clinical populations [33]. Combined, these effects could contribute to a heighted state of negative affect and antagonistic physiological ‘set’ that has been associated with poor outcome or adverse events in prior studies [34]. Interestingly, both insomnia and hypersomnia were associated with reduced likelihood of remission. Superficially, these findings may seem paradoxical, and challenging to reconcile with the more widely disseminated notion that insufficient sleep is a causal factor in the exacerbation of depressive symptoms. However, the acknowledged U-shaped relationship between sleep duration and daytime functioning, [35, 36], as well as known risk for medical and psychiatric comorbidities [37] lends further support to nature of the relationships we observed. Although the literature regarding the relationship between hypersomnia and depressive symptom severity is less mature in comparison, hypersomnia and/or excessive sleepiness may be a sign of homeostatic sleep dysregulation [38] or non-restorative sleep consequently prolonging time-in-bed and delayed rise times to recover sleep debt [39]. This aligns with the well-established body of work linking homeostatic dysregulation of sleep pressure with less favourable treatment outcomes in depression [40–42].

### Sleep as an Endophenotype; Implications for Precision Medicine Approaches to Psychedelics

An alternative explanation is offered by the possibility that differences in sleep symptomology may reflect different phenotypic variants in which sleep disturbances are common but may not necessarily be involved in psilocybin’s mechanism of action. By example, it is well known that around 13% [43] of individuals with depression present with a primary sleep complaint of hypersomnia, rather than one of insomnia (often referred to as ‘*Atypical Depression’*), illustrating that distinct sleep-related phenotypes exist amongst the depressed population. However, it is indeed plausible that the presence of hypersomnia versus insomnia may represent markers of different phenotypes characterized by latent constructs which share a common etiology (e.g., personality dimensions, genetics), and operate external to our predictive models, hence we caution against an inference of causality from these findings. More importantly, as we did not recruit treatment-seeking participants, and only collected self-report diagnoses and mental health symptoms, further studies are warranted amongst ADOPT-participants with mood disorders and documented diagnostic history. Therefore, the symptom trajectories we present should not be considered a clinical outcome. Furthermore, by using the QIDS sleep sub-scale as our outcome, all sleep symptoms in our longitudinal models were compounded, and we are unable to differentiate symptom specific effects. Given these observations, our findings suggest that low-burden, self-report measures of sleep could be used to differentiate the trajectories of responders and non-responders, disregarding any diagnoses. Assuming further replication and confirmation of specificity, advancements in these relatively simple methods could lead to viable methods of selecting suitable candidates for those seeking depressive symptom improvements from ADOPT.

### Should Sleep be a Therapeutic Target in Psychedelic Medicine?

Based on the data we present here, reductions in sleep-disturbance were observed amongst participants following psilocybin administration. It logically follows that psilocybin may hold hypnogenic properties, and an expansion of ADOPT’s experimental indications to include insomnia disorder could be warranted. However, it should be noted that the magnitude of this observed difference was small when measured amongst the entire cohort, both relative to depressive symptom improvements and when compared with existing first-line sleep interventions [44], although sensitivity analyses demonstrated that participants who reported moderate to severe sleep disturbances demonstrated more substantial improvements in sleep (d =-0.45). Furthermore, the conclusions which may reasonably be drawn from these findings require a tentative balance between assertions of causality and bidirectionality. Nonetheless, our finding that sleep significantly predicted subsequent improvements in depression, whereas depression did not predict sleep changes, provides some support in surmounting the limitations posed by our non-randomized design. Notwithstanding the question of directionality or causality, it remains that the four QIDS items we used to assess sleep disturbances fail to capture several constructs prominent in insomnia disorder, such as sleep-related anxiety, daytime dysfunction, and chronicity. Therefore, future assessments are required to determine whether these findings extend to clinical insomnia disorder, using instruments validated for the assessment of sleep disorders.

### Strengths and Limitations

Our findings should only be interpreted within the context of the study’s limitations, perhaps the most conspicuous of which is the lack of a control group, which limits our ability to isolate causal treatment effects. Second, our naturalistic sample gave rise to heterogeneity in dose and dosing-protocol, retreat-location, and population amongst others. The naturalistic setting imposes further limitations, when considering that the fact that retreats may have occurred in destinations other than the participants home-country, meaning the confound of time-zone travel is difficult to account for. Furthermore, although a significant proportion of our sample demonstrated clinically-significant depressive symptoms, we did not perform diagnostic screening, nor did we attempt to recruit a sample with an existing diagnosis of mood disorders, which makes transference to these highly relevant populations difficult. Several prior studies have already demonstrated a relationship between greater self-report sleep disturbance at baseline and poorer depressive symptom outcome amongst patients receiving antidepressant interventions [40–42]. However, many of these studies included sleep symptoms in their depression outcomes and failed to account for the confounding role of other depressive symptoms, and their complex bidirectional relation to sleep disturbance. Our analytic approach, on the other hand, has the distinct advantage of explicitly controlling for indirect and direct pathways between symptoms within these models, increasing our confidence in interpretations made about the relationship between individual symptoms and outcome.

## Conclusions

In conclusion, the literature base regarding psilocybin’s effects on sleep is limited. Nevertheless, our preliminary evidence, demonstrated across multiple rigorous models, suggests a highly complex yet prominent role of sleep in psilocybin’s therapeutic antidepressant action. Whilst the precise mechanism or causal nature of this relationship remains unclear, it is certainly apparent that sleep represents a promising investigational target that is worthy of concerted empirical attention. Subsequent work in this arena could benefit from extending our methodology to randomized controlled designs amongst samples with diagnosed mood disorders and sleep disorders. Such work has credible prospects of yielding more effective and personalized treatment strategies for individuals undergoing ADOPT across a spectrum of conditions.

## Supplementary Information

Below is the link to the electronic supplementary material.

Supplementary file1 (DOCX 7989 KB)

## Data Availability

The data that support the findings of this study are not openly available due to reasons of sensitivity and are available from the corresponding author upon reasonable request. Data are located in controlled access data storage at Imperial College London.
