# Correction to: Preliminary Evidence of Sleep Improvements Following Psilocybin Administration, and their Involvement in Antidepressant Therapeutic Action

**DOI:** 10.1007/s11920-026-01685-1

**Published:** 2026-06-05

**Authors:** Matthew J. Reid, Hannes Kettner, Tessa F. Blanken, Brandon Weiss, Robin Carhart-Harris

**Affiliations:** 1 Behavioral Sleep Medicine Research Laboratory, Department of Psychiatry and Behavioral Sciences, Johns Hopkins School of Medicine, 5510 Nathan Shock Drive, Suite 100, Baltimore, MD 21224 USA; 2 Johns Hopkins Precision Medicine Center of Excellence (PMCoE) for Depression, Johns Hopkins Hospital, Baltimore, MD USA; 3 Department of Neurology, University of California San Francisco, San Francisco, CA USA; 4 Department of Psychological Methods and Clinical Psychology, University of Amsterdam, Amsterdam, Netherlands; 5 Center for Psychedelics and Consciousness Research, Department of Psychiatry and Behavioral Sciences, Johns Hopkins School of Medicine, Baltimore, MD USA; 6 Department of Medicine, Centre for Psychedelic Research, Imperial College London, London, UK

**Correction to: Current Psychiatry Reports (2024) 26:659–669**

10.1007/s11920-024-01539-8

In the original version of this article, the author's name was misspelled as 'Carhartt-Harris.' The correct spelling is 'Carhart-Harris'.

The original article has been corrected.

