# Supplementary material for: Preliminary Evidence of Sleep Improvements Following Psilocybin Administration, and their Involvement in Antidepressant Therapeutic Action
Source: Curr Psychiatry Rep. 2024 Nov 13;26(11):659–69. doi: 10.1007/s11920-024-01539-8 (PMC11579049; doi:10.1007/s11920-024-01539-8)
Supplement: Supplementary file 1 — Supplementary file1 (DOCX 7989 KB) [file 11920_2024_1539_MOESM1_ESM.docx]

**Supplementary Data File**

**Supplementary Table 1a: Baseline Demographics**

|  | **Mean** | | **SD** | | **Median** | | **Min** | **Max** | | | **Skew** | | | **Kurtosis** | | | | | **SE** | | | |  |  |  |  |
| --- | --- | --- | --- | --- | --- | --- | --- | --- | --- | --- | --- | --- | --- | --- | --- | --- | --- | --- | --- | --- | --- | --- | --- | --- | --- | --- |
| Sleep scale baseline | 1.802 | 0.802 | | 2 | | 0 | | | 3 | | | -0.601 | | | | 0.105 | | | | | 0.029 | | | | |  |
| Appetite Weight Scale baseline | 0.630 | 0.854 | | 0 | | 0 | | | 3 | | | 0.780 | | | | 0.030 | | | | | 0.030 | | | | |  |
| Psychomotor Scale bl | 0.695 | 0.825 | | 0 | | 0 | | | 3 | | | 0.205 | | | | 0.029 | | | | | 0.029 | | | | |  |
| QIDS total baseline | 6.778 | 4.667 | | 5 | | 0 | | | 24 | | | 0.495 | | | | 0.168 | | | | | 0.168 | | | | |  |
| QIDS total baseline (without sleep items) | 4.976 | 4.452 | | 4 | | 0 | | | 21 | | | 1.055 | | | | 0.520 | | | | | 0.1611 | | | | |  |
| Income (USD) | 113420 | 4 | | 10000 | | 0 | | | 6mn | | | 27.37 | | | | 749 | | | | | 79160 | | | | |  |
| Age | 44.632 | 12.752 | | 44 | | 19 | | | 76 | | | 0.257 | | | | -0.752 | | | | | 0.461 | | | | |  |
| Dose | 2.3036 | 0.635 | | 2 | | 1 | | | 3 | | | -0.354 | | | | -0.700 | | | | | 0.029 | | | | |  |
| Retreat Days | 3.518 | 3.175 | | 3 | | 0 | | | 15 | | | 0.589 | | | | 0.066 | | | | | 0.115 | | | | |  |
| Number of Ceremonies | 1.859 | 1.315 | | 1 | | 1 | | | 15 | | | 26.611 | | | | 0.047 | | | | |  | | | | |  |
| Gender | Male: 428 | | | |  | |  | | |  | | |  | | | |  | | | | |  | |  |  |  |
|  | Female: 329 | | | |  | |  | | |  | | |  | | | |  | | | | |  | |  |  |  |
|  | Other: 5 | | | |  | |  | | |  | | |  | | | |  | | | | |  | |  |  |  |
| Nationality | US - United States :332 | | | | | |  | | |  | | |  | | | |  | | | | |  | |  |  |  |
|  | GB - United Kingdom:145 | | | | | |  | | |  | | |  | | | |  | | | | |  | |  |  |  |
|  | AU – Australia: 31 | | | |  | |  | | |  | | |  | | | |  | | | | |  | |  |  |  |
|  | DE - Germany: 28 | | | |  | |  | | |  | | |  | | | |  | | | | |  | |  |  |  |
|  | CA - Canada: 25 | | | |  | |  | | |  | | |  | | | |  | | | | |  | |  |  |  |
|  | NL - Netherlands: 18 | | | |  | |  | | |  | | |  | | | |  | | | | |  | |  |  |  |
|  | (Other): 184 | | | | | |  | | |  | | |  | | | |  | | | | |  | |  |  |  |
| Education | None: 5 | | | | | |  | | |  | | |  | | | |  | | | | |  | |  |  |  |
|  | High School Diploma: 54 | | | |  | |  | | |  | | |  | | | |  | | | | |  | |  |  |  |
|  | Associate degree/Technical Degree: 55 | | | | | | | | | | | | | | | | | | | | | | | | | |
|  | College Diploma :233 | | | | | | | | | | | | | |  | | |  | |  | | |  | | | |
|  | Master’s Degree: 253 | | | | | | | | | | | | | |  | | |  | |  | | |  | | | |
|  | Doctorate or Professional Degree (e.g., MD, PhD, Law Degree, JD):162 | | | | | | | | | | | | | |  | | |  | |  | | |  | | | |
|  | Never married :234 | | | | | | | | | | | | | |  | | |  | |  | | |  | | | |
|  | Cohabiting with partner: 98 | | | | | | | | | | | | | |  | | |  | |  | | |  | | | |
|  | Married:318 | | | | | | | | | | | | | |  | | |  | |  | | |  | | | |
|  | Separated: 28 | | | | | | | | | | | | | |  | | |  | |  | | |  | | | |
|  | Divorced: 75 | | | | | | | | | | | | | |  | | |  | |  | | |  | | | |
| Employment | Student (Part-time): 11 | | | | | | | | | | | | | |  | | |  | |  | | |  | | | |
|  | Student (Full time): 31 | | | | | | | | | | | | | |  | | |  | |  | | |  | | | |
|  | Part-time job: 114 | | | | | | | | | | | | | |  | | |  | |  | | |  | | | |
|  | Full-time job :487 | | | | | | | | | | | | | |  | | |  | |  | | |  | | | |
|  | Retired: 66 | | | | | | | | | | | | | |  | | |  | |  | | |  | | | |
|  | White :677 | | | | | | | | | | | | | |  | | |  | |  | | |  | | | |
|  | Prefer not to say: 18 | | | | | | | | | | | | | |  | | |  | |  | | |  | | | |
|  | Asian: 45 | | | | | | | | | | | | | |  | | |  | |  | | |  | | | |
|  | Black or African American: 8 | | | | | | | | | | | | | |  | | |  | |  | | |  | | | |
|  | Unknown: 11 | | | | | | | | | | | | | |  | | |  | |  | | |  | | | |
|  | American Indian or Alaskan Native: 3 | | | | | | | | | | | | | |  | | |  | |  | | |  | | | |
| Diagnoses | MDD: 97 | | | | | | | | | | | | | |  | | |  | |  | | |  | | | |
|  | BPAD: 19 | | | | | | | | | | | | | |  | | |  | |  | | |  | | | |
|  | GAD: 111 | | | | | | | | | | | | | |  | | |  | |  | | |  | | | |
|  | AUD: 10 | | | | | | | | | | | | | |  | | |  | |  | | |  | | | |
|  | SUD: 21 | | | | | | | | | | | | | |  | | |  | |  | | |  | | | |
|  | Schizophrenia: 1 | | | | | | | | | | | | | |  | | |  | |  | | |  | | | |
|  | Psychosis: 2 | | | | | | | | | | | | | |  | | |  | |  | | |  | | | |
|  | Eating Disorder: 15 | | | | | | | | | | | | | |  | | |  | |  | | |  | | | |
|  | Personality Disorder: 16 | | | | | | | | | | | | | |  | | |  | |  | | |  | | | |
|  | Hallucination Persisting Perception Disorder: 5 | | | | | | | | | | | | | |  | | |  | |  | | |  | | | |

**Supplementary Table 1b;** **Individual QIDS Item scores at baseline**

| **QIDS item** | **Mean** | **SD** | **Median** | **Min** | **Max** | **Skew** | **Kurtosis** | **SE** |
| --- | --- | --- | --- | --- | --- | --- | --- | --- |
| Falling asleep Baseline | 0.604 | 0.734 | 0.000 | 0.000 | 2.000 | 0.771 | -0.769 | 0.027 |
| Sleep during the night | 1.570 | 0.944 | 2.000 | 0.000 | 3.000 | -0.408 | -0.803 | 0.034 |
| Waking up too early | 0.541 | 0.899 | 0.000 | 0.000 | 3.000 | 1.539 | 1.206 | 0.033 |
| Sleeping too much | 0.290 | 0.511 | 0.000 | 0.000 | 3.000 | 1.587 | 1.964 | 0.019 |
| Feeling sad | 0.912 | 0.830 | 1.000 | 0.000 | 3.000 | 0.687 | -0.053 | 0.030 |
| Decreased appetite | 0.153 | 0.408 | 0.000 | 0.000 | 3.000 | 8.371 | 0.015 | 0.015 |
| Increased appetite | 0.245 | 0.643 | 0.000 | 0.000 | 3.000 | 2.917 | 8.218 | 0.023 |
| Decreased weight Within the Last Two Weeks | 0.228 | 0.557 | 0.000 | 0.000 | 3.000 | 2.711 | 7.551 | 0.020 |
| Increased weight Within the Last Two Weeks | 0.253 | 0.613 | 0.000 | 0.000 | 3.000 | 2.594 | 6.377 | 0.022 |
| Concentration decision making | 0.722 | 0.764 | 1.000 | 0.000 | 3.000 | 0.568 | -0.910 | 0.028 |
| View of myself | 0.769 | 1.101 | 0.000 | 0.000 | 3.000 | 1.189 | -0.091 | 0.040 |
| Thoughts of death or suicide | 0.308 | 0.632 | 0.000 | 0.000 | 3.000 | 2.132 | 4.103 | 0.023 |
| General interests | 0.459 | 0.759 | 0.000 | 0.000 | 3.000 | 1.675 | 2.173 | 0.027 |
| Energy level | 0.480 | 0.699 | 0.000 | 0.000 | 3.000 | 1.257 | 0.689 | 0.025 |
| Feeling slowed down | 0.236 | 0.517 | 0.000 | 0.000 | 3.000 | 2.240 | 4.734 | 0.019 |
| Feeling restless | 0.417 | 0.720 | 0.000 | 0.000 | 3.000 | 1.859 | 3.135 | 0.026 |

Variable diagnostics and missingness assessments

We first performed descriptive diagnostic tests on variables to assess the degrees of missingness, outlier behavior and central tendency. The outputs of these tests are provided in supplementary files B and C.


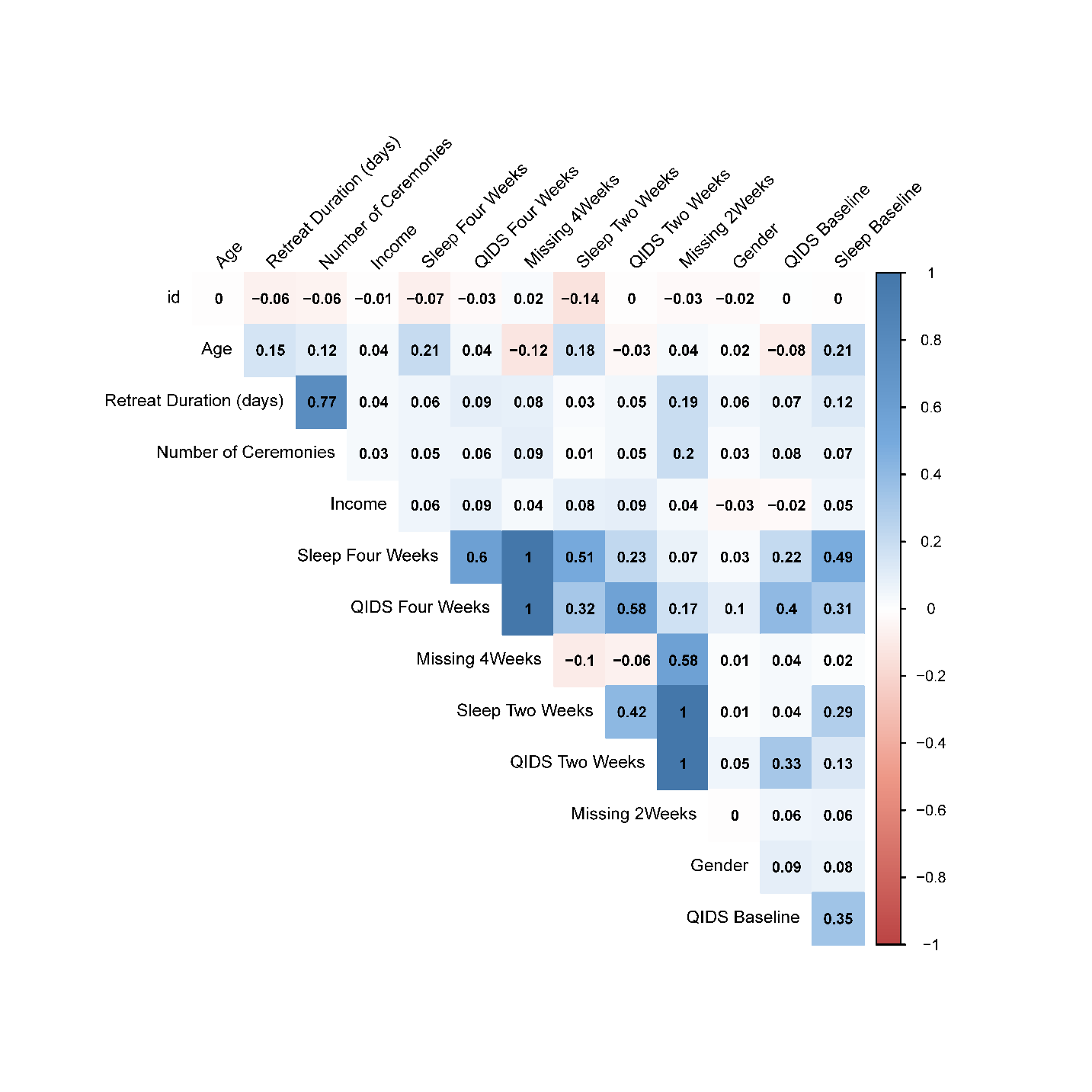


**Supplementary Figure 1: Zero-Order Correlation Matrix of Variables and Covariates**

### Mixed-effects models – Detailed Methods

Outcomes/Response Variables were change in QIDS-depression total score and QIDS-sleep total score at two weeks and four weeks. Fixed effects in both models included timepoint

(baseline, two-weeks, four-weeks), and the respective outcome measure at baseline to account for potential confounding of initial symptom severity (outcome ~ 1 + outcome at baseline + timepoint + [1 | ID]). We performed model diagnostics, by visually assessing the normality and skedasticity of residuals. Assumptions were also evaluated by comparing variance components from null and conditional models, according to Singer and Willett ^41^. Through this process, maximum likelihood estimate was used to evaluate model fit. We based our primary outcomes and interpretations of findings on unadjusted models, to optimise model parsimony and facilitate hypothesis generation, and to aid in comparison with future studies, given this early exploratory stage. However, we conducted pre-planned sensitivity analyses to incrementally adjust for and assess the impact of available covariates (Age, ethnicity, marital status, employment, income, country of retreat, number of sessions, psilocybin dose, retreat-center, retreat-duration) on model fit and interpretation, which we report in full in the table (below).

**Supplementary Table 2**: **Incremental Adjustments** Values represent non-bootstrapped coefficients of first -order adjusted models. Models were extracted from the recursive covariate selection model which tested the introduction of 1^st^ to K order covariates, and all of the possible permutations of the 6 covariates (Marital Status, Income, Education, Gender, Center Number, Ethnicity/Race, Number of Ceremonies/ Doses of Psilocybin). Full models are included in supplementary file A. Abbreviations: bl = baseline, ID = participant ID

| **Covariate** | **Estimate** | **SE** | **T val** | **P val** | **AIC** | **BIC** | **REML** | **Formula** |  |
| --- | --- | --- | --- | --- | --- | --- | --- | --- | --- |
| Marital | -0.140 | 0.026 | -5.324 | 1.43E-07 | 1369.039 | 1396.663 | 1357.039 | sleep ~ 1 + Marital + sleep_bl + time + (1 \| ID) | |
| Income | -0.135 | 0.026 | -5.132 | 3.85E-07 | 1397.353 | 1424.993 | 1385.353 | sleep ~ 1 + Income + sleep_bl + time + (1 \| ID) | |
| Education | -0.134 | 0.026 | -5.096 | 4.63E-07 | 1381.321 | 1408.985 | 1369.321 | sleep ~ 1 + Education + sleep_bl + time + (1 \| ID) | |
| Gender | -0.134 | 0.026 | -5.095 | 4.63E-07 | 1380.077 | 1407.741 | 1368.077 | sleep ~ 1 + Gender + sleep_bl + time + (1 \| ID) | |
| Center | -0.133 | 0.026 | -5.069 | 5.29E-07 | 1387.674 | 1415.338 | 1375.674 | sleep ~ 1 + Center + sleep_bl + time + (1 \| ID) | |
| Ethnicity | -0.134 | 0.026 | -5.066 | 5.37E-07 | 1384.801 | 1426.297 | 1366.801 | Sleep ~ 1 + Ethnicity + sleep_bl + time + (1 \| ID) | |
| Number of Ceremonies | -0.133 | 0.026 | -5.039 | 6.16E-07 | 1381.048 | 1408.712 | 1369.048 | Sleep ~ 1 + NumCers + sleep_bl + time + (1 \| ID) | |

Given that large variations in sample size can give rise to spurious fixed effects in nested random-effects models, we also performed a Bayesian-simulation model using Montecarlo Markov Chains to generate robust estimates of expected variation in our two outcome variables (sleep and depressive symptoms) across the range of center sample sizes (Supplementary figure 2), to distinguish any meaningful center-based effects from natural variance.


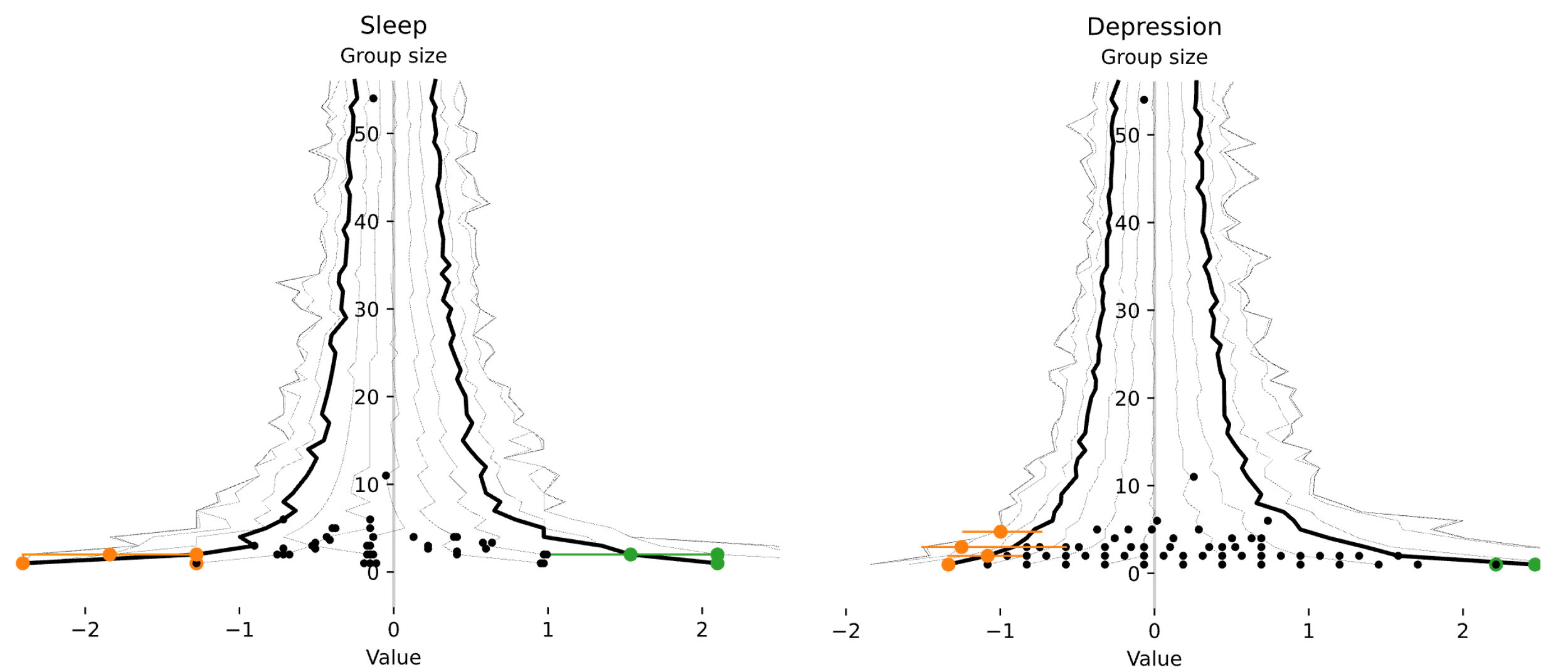


**Supplementary Figure 2:** **Funnel plot of observed/expected variation for sleep and depressive symptom improvements at week 4, (z-scored slope) by center sample size.** Solid black lines represent the distribution of expected variance (95% Bayesian credible intervals) in the outcomes according to the sample size of the contributing center. Credible intervals were generated from 2000 iterations of Montecarlo Markov Chain simulations (4 chains). Centers falling below (orange) or above (green) the 95% credible interval demonstrated marginally greater than expected variance in the outcome measures suggesting small but significant differences in outcome between centers.

Resampling procedures were performed on the uncorrected, and best-fitting corrected models to obtain estimates of the stability of the 95% Confidence Interval (CI) of the fixed-effect coefficients. To optimise the certainty of these CIs amongst high instances of missing data (53-55%), we performed supervised recursive bootstrapping(Rohekar, Gurwicz et al. 2018) for each model which assessed when convergence criteria (convergence threshold = 1$\varepsilon$-3) were met and increasing the number of iterations (by 500 each cycle) did not result in any further increase in model performance. Univariate normality was assessed descriptively by skew > |3| or kurtosis > |3| considered problematic. Cook’s distance was calculated for each model to identify observations that influence the model results (i.e., leverage points). Models were analysed both with and without leverage points included in the model to understand whether the results are influenced by a few unique cases. Models without leverage points were interpreted.

Muliple imputation testing

Missing outcome data at each two weeks (53%) and four-week (55%) were determined Missing Not at Random (MNAR), as greater observed values for: age, number of sessions, duration of retreat, and outcome at baseline were significantly associated with increased missingness. Random effects models are implicitly robust to missing data(Peters, Bots et al. 2012), therefore we did not perform listwise deletion and based our interpretations on unimputed data. However, given the large degree of missing data, we performed sensitivity analyses to evaluate whether two methods of imputation influenced our findings. Method 1 involved a ‘worst-case scenario’ imputation using Baseline-Observation-Carried-Forward (BOCF), where missing values were replaced with each participant’s baseline value, under the assumption that any change from baseline has regressed to pre-study levels. This method is preferred to other single imputation methods, such as Last Observation Carried Forward (LOCF), in instances where loss to follow up is not missing at random(Helms, Reece et al. 2011). Method 2 involved a less conservative Multiple Imputations via Chained Equations (MICE) method, whereby multiple randomly imputed datasets are generated, statistical models are then performed on each dataset, and the results of each model are pooled. MICE using predictive mean matching or maximum likelihood estimation is the preferred method of imputation and has shown to demonstrate superior classification accuracy compared with single imputation, in instances of data missing at random, with up to 50% missingness(Mera-Gaona, Neumann et al. 2021). To address the complexity of large proportions of missing data, and the pattern of MNAR in our dataset, we implemented a neural-network driven deep-learning model, applied to Multiple Imputation using Chained Equations (MICE), which has previously demonstrated clear superiority of prediction accuracy and classification error in MNAR data in instances of up to 80% missingness (Lall and Robinson 2021). We generated 100 imputation datasets, and compared fit and performance between the unimputed models, and models imputed using Method 1 and Method 2 by comparing the Restricted Maximum Likelihood (REML) and Bayesian Information Criterion (BIC) values. using Method 1 and Method 2 by comparing the Restricted Maximum Likelihood (REML) and Bayesian Information Criterion (BIC) values. These effects were also sustained following sensitivity analyses, remaining significant after 1): being performed on the dataset imputed using multiple imputations via deep learning (β = -0.16 [^SE^0.02], 95% CI: -0.08 to -0.01, p = <0.001, d = -0.35) and also ‘worst-case’ imputation using BOCF (β = -0.01 [^SE^0.01], 95% CI: -0.08 to -0.01, p = 0.016, d = -0.04).


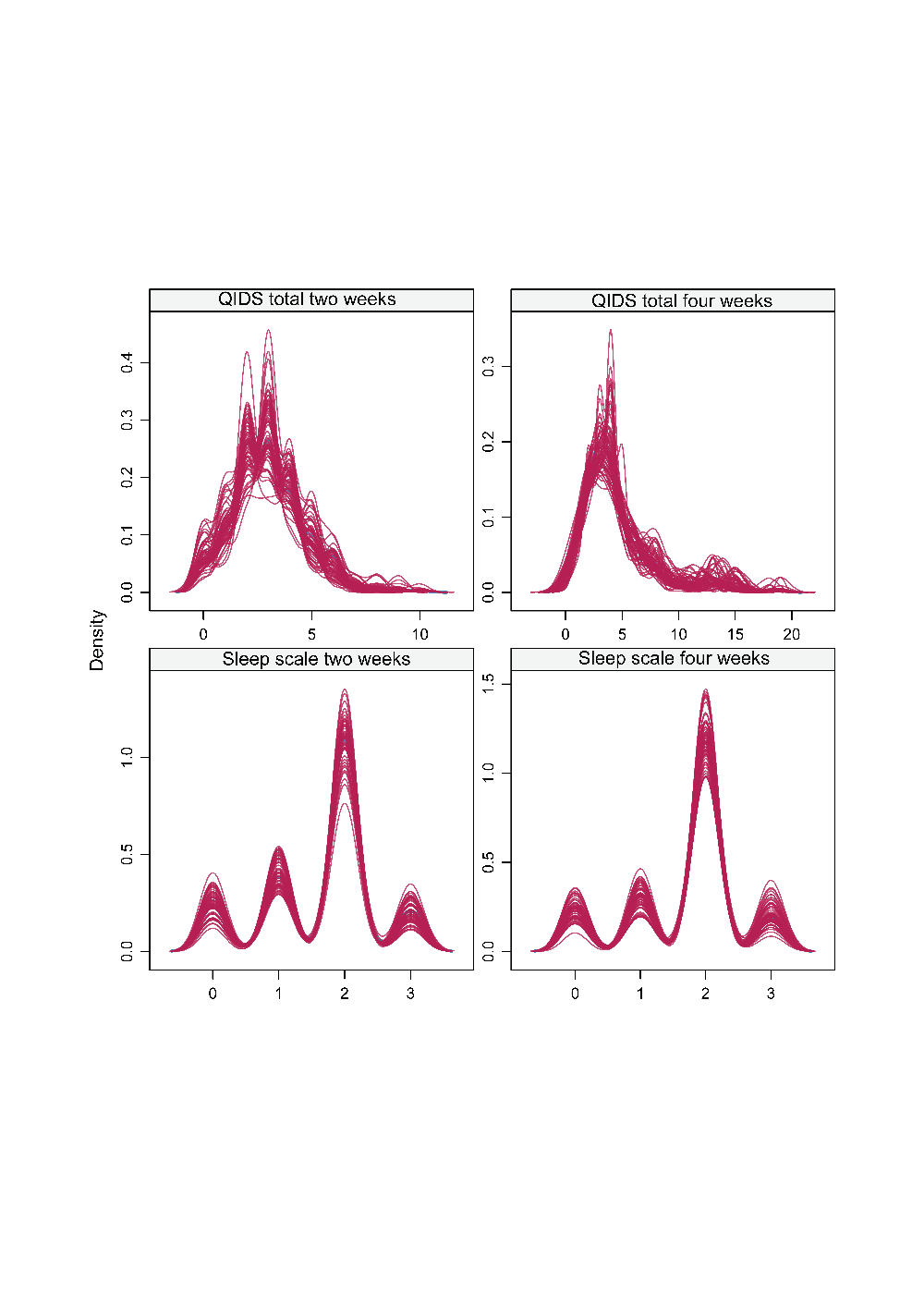


**Figure 6: Imputation models**. Figures demonstrate outcome of 100 imputed datasets using Multiple Imputations vis Chained Equations.

Imputation Models Fit Indices

|  | Bayesian Information Criterion | Akaike Information Criterion | REML |
| --- | --- | --- | --- |
| Index | 1396.663 | 1360.039 | 1357.039 |
| MICE | 1396.886 | 1371.810 | 1363.813 |
| BOCF | 1409.059 | 1376.882 | 1362.832 |

Supplementary Table 3

|  | Subgroup |  | Estimate | SE | t value | P value | cohens d | estimate | Lower CI | Upper CI |
| --- | --- | --- | --- | --- | --- | --- | --- | --- | --- | --- |
| QIDS-depression | (QIDS > 5) | (Intercept) | 1.883 | 0.108 | 17.420 | <2e-16 | 0.452 | 1.883 | 1.690 | 2.071 |
|  |  | Baseline | 0.622 | 0.014 | 45.080 | <2e-16 | 1.171 | 0.622 | 0.597 | 0.648 |
|  |  | Two weeks | -2.241 | 0.146 | -15.330 | <2e-16 | -0.398 | -2.241 | -2.526 | -1.940 |
|  |  | Four Weeks | -3.501 | 0.148 | -23.600 | <2e-16 | -0.613 | -3.501 | -3.794 | -3.198 |
|  | QIDS >0 | (Intercept) | 3.727 | 0.326 | 11.450 | <2e-16 | 0.639 | 1.883 | 1.690 | 2.071 |
|  |  | Baseline | 0.626 | 0.030 | 21.150 | <2e-16 | 1.283 | 0.622 | 0.597 | 0.648 |
|  |  | Two weeks | -5.954 | 0.241 | -24.740 | <2e-16 | -1.206 | -2.241 | -2.526 | -1.940 |
|  |  | Four Weeks | -7.902 | 0.247 | -32.000 | <2e-16 | -1.544 | -3.501 | -3.794 | -3.198 |
| QIDS-sleep | (QIDS > 0) | (Intercept) | 0.527 | 0.041 | 12.794 | < 2e-16 | 0.406 | 0.527 | 0.440 | 0.611 |
|  |  | Baseline | 0.707 | 0.020 | 36.065 | < 2e-16 | 1.278 | 0.707 | 0.670 | 0.749 |
|  |  | Two weeks | -0.089 | 0.036 | -2.469 | 0.014 | -0.071 | -0.089 | -0.166 | -0.018 |
|  |  | Four Weeks | -0.120 | 0.037 | -3.265 | 0.001 | -0.094 | -0.120 | -0.192 | -0.046 |
|  | QIDS >2 | (Intercept) | 0.591 | 0.093 | 6.334 | 0.000 | 0.249 | 0.591 | 0.413 | 0.739 |
|  |  | Baseline | 0.734 | 0.041 | 17.935 | < 2e-16 | 0.719 | 0.734 | 0.668 | 0.809 |
|  |  | Two weeks | -0.408 | 0.037 | -11.158 | < 2e-16 | -0.380 | -0.408 | -0.479 | -0.347 |
|  |  | Four Weeks | -0.365 | 0.037 | -9.878 | < 2e-16 | -0.336 | -0.365 | -0.444 | -0.289 |

Supplementary Table 4

|  | Outcome |  | Estimate | SE | t value | P value | cohens d | estimate | Lower CI | Upper CI | Estimate |
| --- | --- | --- | --- | --- | --- | --- | --- | --- | --- | --- | --- |
| MIDAS | SLEEP | (Intercept) | 1.487 | 0.126 | 11.796 | <2e-16 | 4.638 | 0.321 | 0.977 | 0.899 | 1.060 |
|  |  | Baseline | 0.781 | 0.013 | 59.860 | <2e-16 | 0.360 | 2.170 | 0.458 | 0.420 | 0.495 |
|  |  | Two weeks | -1.647 | 0.115 | -14.360 | <2e-16 | 4.478 | -0.368 | -0.165 | -0.235 | -0.093 |
|  |  | Four Weeks | -1.084 | 0.115 | -9.448 | <2e-16 | 4.478 | -0.242 | -0.127 | -0.198 | -0.066 |
|  | QIDS | (Intercept) | 3.940 | 0.132 | 29.930 | <2e-16 | 6.293 | 0.626 | 3.940 | 3.692 | 4.207 |
|  |  | Baseline | 0.419 | 0.012 | 33.550 | <2e-16 | 0.597 | 0.702 | 0.419 | 0.394 | 0.443 |
|  |  | Two weeks | -2.482 | 0.143 | -17.410 | <2e-16 | 6.817 | -0.364 | -2.482 | -2.747 | -2.214 |
|  |  | Four Weeks | -3.810 | 0.143 | -26.720 | <2e-16 | 6.817 | -0.559 | -3.810 | -4.081 | -3.548 |
| BOCF | SLEEP | (Intercept) | 0.354 | 0.031 | 11.319 | <2e-16 | 1.043 | 0.340 | 0.354 | 0.283 | 0.415 |
|  |  | Baseline | 0.804 | 0.014 | 56.049 | <2e-16 | 0.396 | 2.031 | 0.804 | 0.777 | 0.835 |
|  |  | Two weeks | -0.052 | 0.023 | -2.265 | 0.024 | 0.904 | -0.058 | -0.052 | -0.094 | -0.007 |
|  |  | Four Weeks | -0.037 | 0.023 | -1.585 | 0.113 | 0.904 | -0.041 | -0.037 | -0.073 | 0.008 |
|  | QIDS | (Intercept) | 1.487 | 0.126 | 11.796 | <2e-16 | 4.638 | 0.321 | 1.487 | 1.199 | 1.739 |
|  |  | Baseline | 0.781 | 0.013 | 59.860 | <2e-16 | 0.360 | 2.170 | 0.781 | 0.753 | 0.812 |
|  |  | Two weeks | -1.647 | 0.115 | -14.360 | <2e-16 | 4.478 | -0.368 | -1.647 | -1.856 | -1.438 |
|  |  | Four Weeks | -1.084 | 0.115 | -9.448 | <2e-16 | 4.478 | -0.242 | -1.084 | -1.284 | -0.841 |
| SLEEP>2 | SLEEP | (Intercept) | 0.591 | 0.093 | 6.334 | 0.000 | 2.372 | 0.249 | 0.591 | 0.413 | 0.739 |
|  |  | Baseline | 0.734 | 0.041 | 17.935 | < 2e-16 | 1.021 | 0.719 | 0.734 | 0.668 | 0.809 |
|  |  | Two weeks | -0.408 | 0.037 | -11.158 | < 2e-16 | 1.074 | -0.380 | -0.408 | -0.479 | -0.347 |
|  |  | Four Weeks | -0.365 | 0.037 | -9.878 | < 2e-16 | 1.088 | -0.336 | -0.365 | -0.444 | -0.289 |

Structural Equation Models

Structural equation modeling was used to examine the dynamic relationships between depressive symptoms (represented as qids in formulae notation) and sleep quality (sleep) across three time points. A cross-lagged panel framework was used to model the variance and covariance where, β_ij_​ and γ_ij_​ represent the coefficients for the QIDS and sleep variables, capturing the lagged events across different time points. Error terms ε-qids​ and ε-sleep​ account for unobserved factors influencing depressive symptoms (qids) and sleep quality (sleep) at each time point. Lagged analyses controlled for the baseline value of the lagged variable (i.e Sleep_0_), as well as the value of the covariate (i.e Depression_2_) at the same timepoint (i.e., Depression^3^ ~ Depression^2^ + Sleep^0^ + Sleep^2^).

Cross-Lagged Panel Models Fit Indices

|  | Standardized Root Mean Square Residuals | Comparative Fit Index | Root Mean Square Error of Approximations | Root Mean Square Error of Approximations (Lower CI) | Root Mean Square Error of Approximations (Upper CI) |
| --- | --- | --- | --- | --- | --- |
| Observed | 0.036 | 0.986 | 0.08 | 0.051 | 0.113 |
| Optimal | ≤0.08 | ≥0.95 | ≤0.08 | - | - |

### Symptom Network Analysis

To investigate symptom-specific relationships between individual QIDS items and overall depressive symptom improvement, we adapted a Network Interventional Analysis(Blanken, Van Der Zweerde et al. 2019) and estimated, for each of the timepoints, an Exponential Mixed Graphical Model(Haslbeck and Waldorp 2020) including each individual QIDS item in addition to a binary outcome variable representing depressive symptom remission.

Network analyses were used to generate exponential mixed graphical models(Yang, Baker et al. 2014) via K-degree nodewise regression. From these models, we calculated the adjusted weights which represent the strength of association between individual contemporaneous symptoms and our lagged remission variable. We then applied a LASSO regularization to determine the extent to which sampling variation may have led to the inclusion of spurious edges (Epskamp and Fried 2018) in accordance with previous recommendations (Epskamp and Isvoranu 2022, Isvoranu and Epskamp 2023). We generated 1000 new resampled networks using the ‘resample’ function of the MGM package, and verified the accuracy and stability of the estimated network models (Epskamp and Fried 2018) by assessing the probability (%) that a given edge (i.e., link between symptoms) was present in the network after resampling, as well as the strength of the included links, by assessing the range of the adjusted edge weights observed during resampling.

In our network analyses, all variables (e.g., the QIDS items and the response variables [remission]) are included as individual nodes and visualised in a network. nodes are linked by edges that represent conditional-dependence among them, i.e., the unique association between two variables after conditioning on all other variables in the network (‘adjusted weights’). Therefore, the relationships between the individual symptoms and remission we report implicitly controls for the confounds of the remaining symptoms. We used the magnitude and direction of adjusted edge weights to interpret the magnitude of these relationships, whereby positive (Increased likelihood of remission) and negative (Decreased likelihood of remission) relationships were represented by blue (positive) and red (negative) edges. Finally, we tested the predictive accuracy of our models using K-Fold Cross-Validation, by dividing the datasets into K (10) non-overlapping datasets (folds). For each of 10 iterations, the model was trained on 9 datasets and we validated the model’s ability to predict values in the remaining dataset.

Our analyses were initially confined to those who demonstrated clinically significant depressive symptoms, as defined as a score >5 on the QIDS total score at baseline, a common cutoff for ‘mild depression’ given the application relevance. For transparency, we also report networks derived from the whole sample (supplementary figures 1-4). We further probed the extent to which any relationships between sleep symptoms and depressive symptom improvement may have been influenced by the presence of sleep items in the outcome variable, after reintroduction of sleep item. We conducted primary analyses unadjusted but performed sensitivity analyses by sequentially introducing additional nodes for study center, psilocybin dose, and the number of sessions to examine their impact on the network relationships demonstrated in our primary analyses.

Sensitivity Networks (Adjusted for Treatment Center)


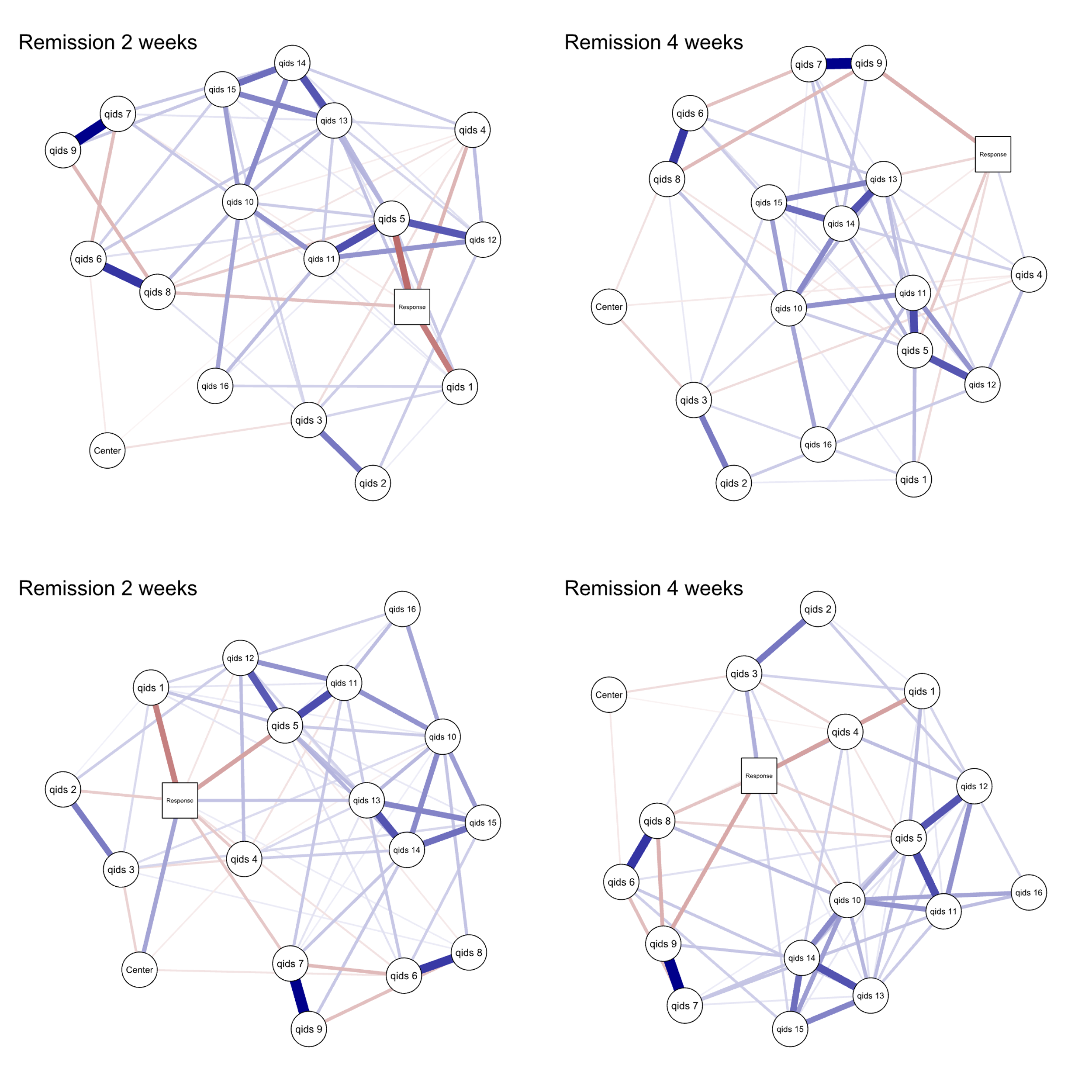


**Supplementary Figure 1:** **Symptom Networks after including study center as a covariate:** Exponential Mixed Graphical Models of Symptom Networks generated by K-degree nodewise regression. In network analyses, a direct link (i.e., an ‘Edge’) between nodes (circles) and the lagged outcome node [‘Remit’/Square] represents a direct relationship between the two variables, after controlling for relationships amongst all other nodes. The thickness and opacity of the edge connecting nodes represents the magnitude of the relationship (thicker = stronger), after accounting for other nodes. Red edges indicated a negative/inverse association between the symptom and remission (i.e., reduced likelihood of remission), whereas blue edges indicate a positive (i.e., increased likelihood of remission) association. Remit = Remission of depressive symptoms at timepoint (QIDS<6). Networks were cross-validated using k-folds trained on 10 datasets. Bottom two panels: Networks after removal of the 4 QIDS sleep items from the QIDS total score, Top two panels: Networks including the 4 QIDS sleep items in the total score.

Dose


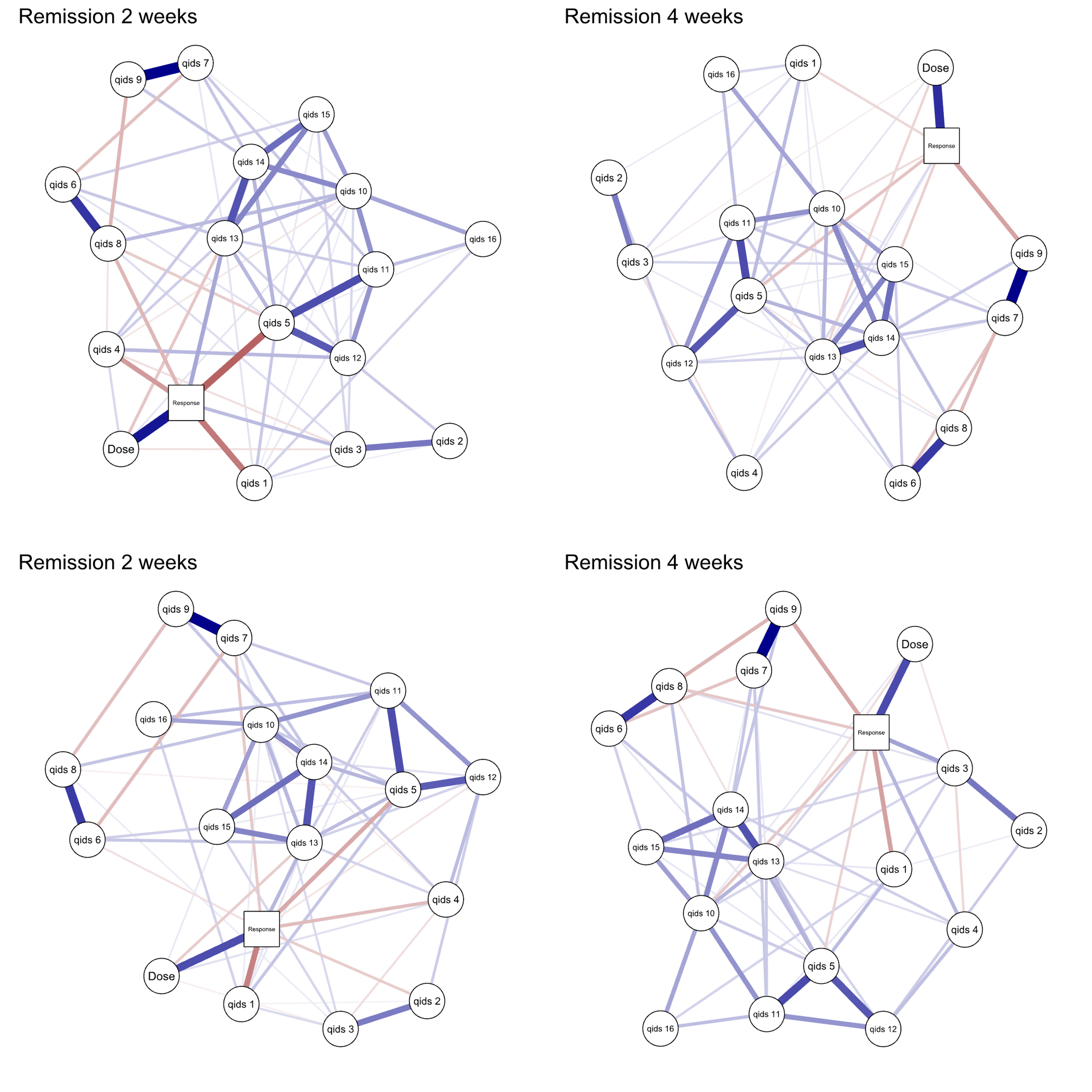


**Supplementary Figure 2**: **Symptom Networks after including psilocybin dose as a covariate:** Exponential Mixed Graphical Models of Symptom Networks generated by K-degree nodewise regression. In network analyses, a direct link (i.e., an ‘Edge’) between nodes (circles) and the lagged outcome node [‘Remit’/Square] represents a direct relationship between the two variables, after controlling for relationships amongst all other nodes. The thickness and opacity of the edge connecting nodes represents the magnitude of the relationship (thicker = stronger), after accounting for other nodes. Red edges indicated a negative/inverse association between the symptom and remission (i.e., reduced likelihood of remission), whereas blue edges indicate a positive (i.e., increased likelihood of remission) association. Remit = Remission of depressive symptoms at timepoint (QIDS<6). Networks were cross-validated using k-folds trained on 10 datasets. Bottom two panels: Networks after removal of the 4 QIDS sleep items from the QIDS total score, Top two panels: Networks including the 4 QIDS sleep items in the total score.

Retreat Duration (Days)


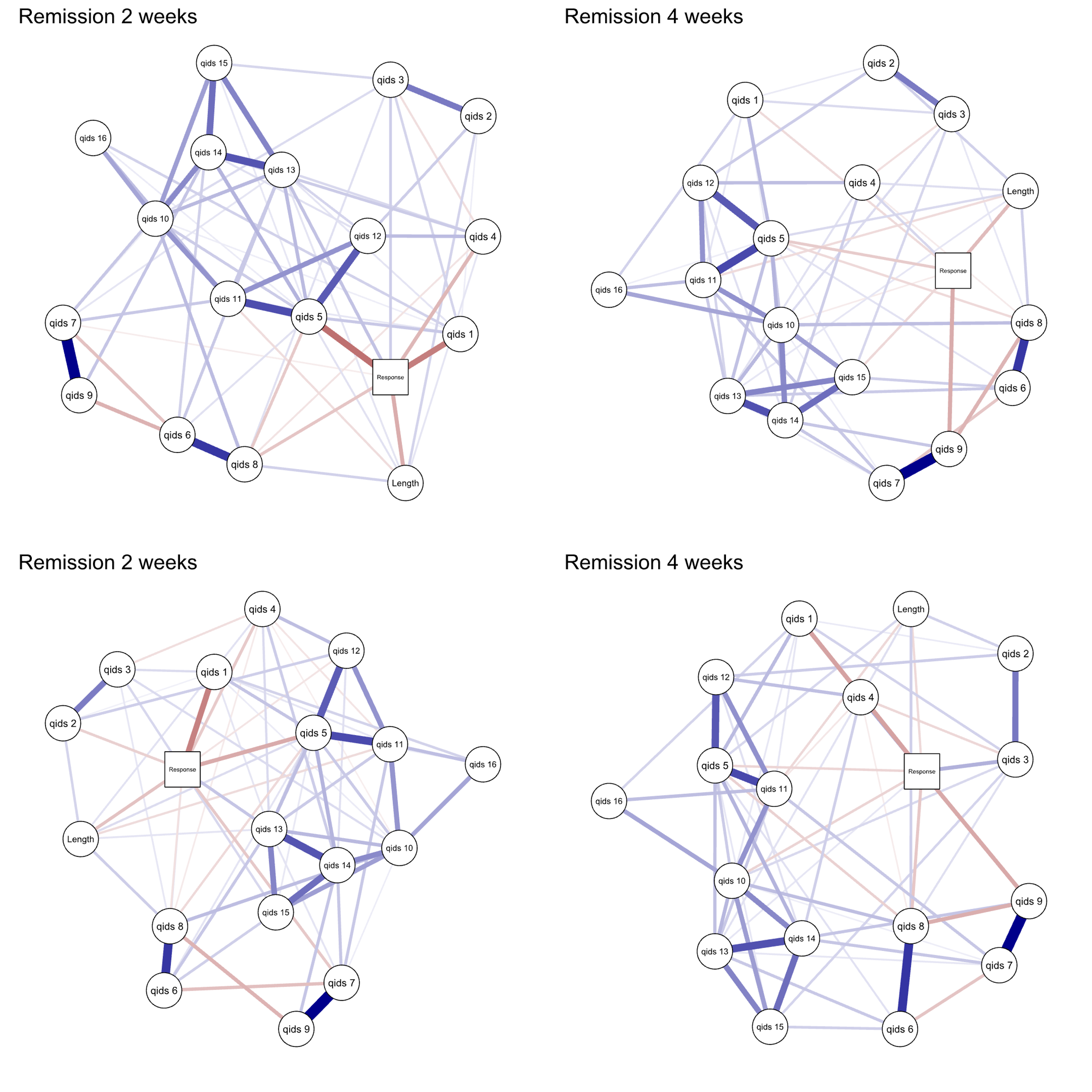


**Supplementary Figure 3:** **Symptom Networks after including length/duration of retreat (days) as a covariate:** Exponential Mixed Graphical Models of Symptom Networks generated by K-degree nodewise regression. In network analyses, a direct link (i.e., an ‘Edge’) between nodes (circles) and the lagged outcome node [‘Remit’/Square] represents a direct relationship between the two variables, after controlling for relationships amongst all other nodes. The thickness and opacity of the edge connecting nodes represents the magnitude of the relationship (thicker = stronger), after accounting for other nodes. Red edges indicated a negative/inverse association between the symptom and remission (i.e., reduced likelihood of remission), whereas blue edges indicate a positive (i.e., increased likelihood of remission) association. Remit = Remission of depressive symptoms at timepoint (QIDS<6). Networks were cross-validated using k-folds trained on 10 datasets. Bottom two panels: Networks after removal of the 4 QIDS sleep items from the QIDS total score, Top two panels: Networks including the 4 QIDS sleep items in the total score.

All covariates combined


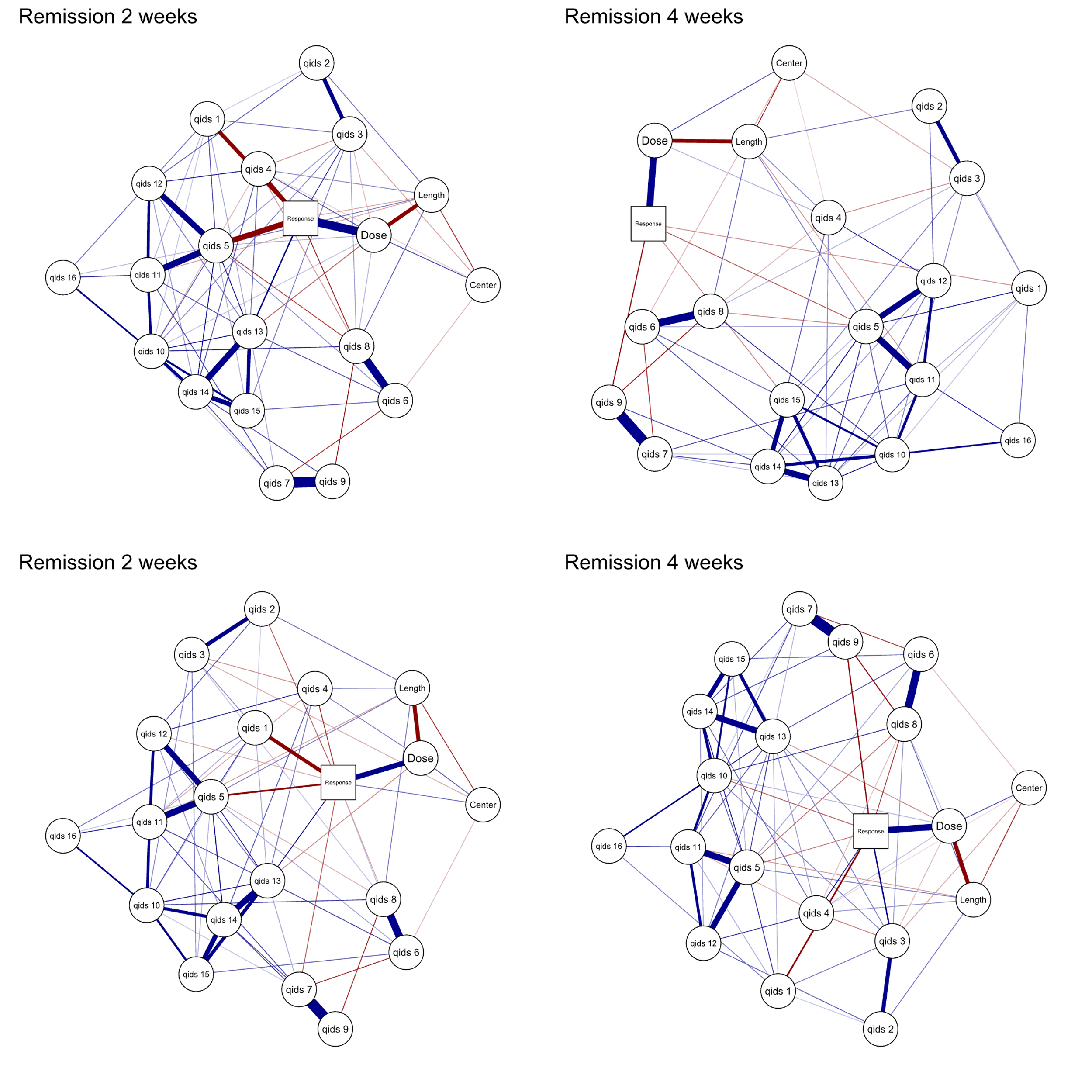


**Supplementary Figure 4:** **Symptom Networks after including dose, study center & length/duration of retreat (days) as a covariate:** Exponential Mixed Graphical Models of Symptom Networks generated by K-degree nodewise regression. In network analyses, a direct link (i.e., an ‘Edge’) between nodes (circles) and the lagged outcome node [‘Remit’/Square] represents a direct relationship between the two variables, after controlling for relationships amongst all other nodes. The thickness and opacity of the edge connecting nodes represents the magnitude of the relationship (thicker = stronger), after accounting for other nodes. Red edges indicated a negative/inverse association between the symptom and remission (i.e., reduced likelihood of remission), whereas blue edges indicate a positive (i.e., increased likelihood of remission) association. Remit = Remission of depressive symptoms at timepoint (QIDS<6). Networks were cross-validated using k-folds trained on 10 datasets. Bottom two panels: Networks after removal of the 4 QIDS sleep items from the QIDS total score, Top two panels: Networks including the 4 QIDS sleep items in the QIDS total score.

Network Weights (Two Weeks)

|  | Remit | QIDS-1 | QIDS-2 | QIDS-3 | QIDS-4 | QIDS-5 | QIDS-6 | QIDS-7 | QIDS-8 | QIDS-9 | QIDS-10 | QIDS-11 | QIDS-12 | QIDS-13 | QIDS-14 | QIDS-15 | QIDS-16 |
| --- | --- | --- | --- | --- | --- | --- | --- | --- | --- | --- | --- | --- | --- | --- | --- | --- | --- |
| Remit |  | 0.145 | 0.000 | 0.119 | 0.122 | 0.068 | 0.000 | 0.000 | 0.092 | 0.135 | 0.069 | 0.000 | 0.000 | 0.032 | 0.000 | 0.000 | 0.000 |
| QIDS-1 | 0.145 |  | 0.031 | 0.070 | 0.000 | 0.106 | 0.000 | 0.000 | 0.000 | 0.000 | 0.000 | 0.038 | 0.000 | 0.038 | 0.000 | 0.000 | 0.069 |
| QIDS-2 | 0.000 | 0.031 |  | 0.212 | 0.000 | 0.000 | 0.000 | 0.000 | 0.000 | 0.000 | 0.000 | 0.000 | 0.080 | 0.000 | 0.000 | 0.000 | 0.000 |
| QIDS-3 | 0.119 | 0.070 | 0.212 |  | 0.063 | 0.000 | 0.000 | 0.000 | 0.052 | 0.000 | 0.062 | 0.000 | 0.000 | 0.000 | 0.000 | 0.066 | 0.000 |
| QIDS-4 | 0.122 | 0.000 | 0.000 | 0.063 |  | 0.000 | 0.000 | 0.000 | 0.000 | 0.000 | 0.000 | 0.000 | 0.102 | 0.050 | 0.077 | 0.000 | 0.000 |
| QIDS-5 | 0.068 | 0.106 | 0.000 | 0.000 | 0.000 |  | 0.045 | 0.000 | 0.065 | 0.000 | 0.082 | 0.282 | 0.264 | 0.102 | 0.117 | 0.037 | 0.000 |
| QIDS-6 | 0.000 | 0.000 | 0.000 | 0.000 | 0.000 | 0.045 |  | 0.096 | 0.318 | 0.000 | 0.000 | 0.000 | 0.000 | 0.086 | 0.000 | 0.075 | 0.000 |
| QIDS-7 | 0.000 | 0.000 | 0.000 | 0.000 | 0.000 | 0.000 | 0.096 |  | 0.000 | 0.400 | 0.029 | 0.086 | 0.000 | 0.041 | 0.089 | 0.000 | 0.000 |
| QIDS-8 | 0.092 | 0.000 | 0.000 | 0.052 | 0.000 | 0.065 | 0.318 | 0.000 |  | 0.124 | 0.103 | 0.000 | 0.000 | 0.000 | 0.000 | 0.000 | 0.000 |
| QIDS-9 | 0.135 | 0.000 | 0.000 | 0.000 | 0.000 | 0.000 | 0.000 | 0.400 | 0.124 |  | 0.000 | 0.000 | 0.000 | 0.000 | 0.090 | 0.000 | 0.000 |
| QIDS-10 | 0.069 | 0.000 | 0.000 | 0.062 | 0.000 | 0.082 | 0.000 | 0.029 | 0.103 | 0.000 |  | 0.170 | 0.000 | 0.112 | 0.186 | 0.153 | 0.141 |
| QIDS-11 | 0.000 | 0.038 | 0.000 | 0.000 | 0.000 | 0.282 | 0.000 | 0.086 | 0.000 | 0.000 | 0.170 |  | 0.167 | 0.081 | 0.000 | 0.000 | 0.102 |
| QIDS-12 | 0.000 | 0.000 | 0.080 | 0.000 | 0.102 | 0.264 | 0.000 | 0.000 | 0.000 | 0.000 | 0.000 | 0.167 |  | 0.069 | 0.049 | 0.000 | 0.000 |
| QIDS-12 | 0.032 | 0.038 | 0.000 | 0.000 | 0.050 | 0.102 | 0.086 | 0.041 | 0.000 | 0.000 | 0.112 | 0.081 | 0.069 |  | 0.271 | 0.193 | 0.000 |
| QIDS-14 | 0.000 | 0.000 | 0.000 | 0.000 | 0.077 | 0.117 | 0.000 | 0.089 | 0.000 | 0.090 | 0.186 | 0.000 | 0.049 | 0.271 |  | 0.229 | 0.000 |
| QIDS-15 | 0.000 | 0.000 | 0.000 | 0.066 | 0.000 | 0.037 | 0.075 | 0.000 | 0.000 | 0.000 | 0.153 | 0.000 | 0.000 | 0.193 | 0.229 |  | 0.000 |
| QIDS-16 | 0.000 | 0.069 | 0.000 | 0.000 | 0.000 | 0.000 | 0.000 | 0.000 | 0.000 | 0.000 | 0.141 | 0.102 | 0.000 | 0.000 | 0.000 | 0.000 |  |

|  | Remit | QIDS-1 | QIDS-2 | QIDS-3 | QIDS-4 | QIDS-5 | QIDS-6 | QIDS-7 | QIDS-8 | QIDS-9 | QIDS-10 | QIDS-11 | QIDS-12 | QIDS-13 | QIDS-14 | QIDS-15 | QIDS-16 |
| --- | --- | --- | --- | --- | --- | --- | --- | --- | --- | --- | --- | --- | --- | --- | --- | --- | --- |
| Remit |  | 0.200 | 0.083 | 0.000 | 0.090 | 0.138 | 0.050 | 0.092 | 0.031 | 0.000 | 0.000 | 0.000 | 0.045 | 0.088 | 0.000 | 0.000 | 0.000 |
| QIDS-1 | 0.200 |  | 0.024 | 0.057 | 0.000 | 0.093 | 0.000 | 0.000 | 0.000 | 0.000 | 0.032 | 0.040 | 0.000 | 0.041 | 0.000 | 0.000 | 0.073 |
| QIDS-2 | 0.083 | 0.024 |  | 0.208 | 0.000 | 0.000 | 0.000 | 0.000 | 0.000 | 0.000 | 0.000 | 0.000 | 0.080 | 0.000 | 0.000 | 0.000 | 0.000 |
| QIDS-3 | 0.000 | 0.057 | 0.208 |  | 0.052 | 0.000 | 0.000 | 0.000 | 0.034 | 0.000 | 0.057 | 0.000 | 0.000 | 0.000 | 0.000 | 0.065 | 0.000 |
| QIDS-4 | 0.090 | 0.000 | 0.000 | 0.052 |  | 0.000 | 0.000 | 0.000 | 0.000 | 0.000 | 0.029 | 0.000 | 0.105 | 0.061 | 0.081 | 0.000 | 0.000 |
| QIDS-5 | 0.138 | 0.093 | 0.000 | 0.000 | 0.000 |  | 0.041 | 0.000 | 0.039 | 0.000 | 0.085 | 0.283 | 0.258 | 0.106 | 0.117 | 0.035 | 0.000 |
| QIDS-6 | 0.050 | 0.000 | 0.000 | 0.000 | 0.000 | 0.041 |  | 0.106 | 0.315 | 0.000 | 0.000 | 0.000 | 0.000 | 0.089 | 0.000 | 0.076 | 0.000 |
| QIDS-7 | 0.092 | 0.000 | 0.000 | 0.000 | 0.000 | 0.000 | 0.106 |  | 0.000 | 0.404 | 0.030 | 0.086 | 0.000 | 0.045 | 0.089 | 0.000 | 0.000 |
| QIDS-8 | 0.031 | 0.000 | 0.000 | 0.034 | 0.000 | 0.039 | 0.315 | 0.000 |  | 0.106 | 0.099 | 0.000 | 0.000 | 0.000 | 0.000 | 0.000 | 0.000 |
| QIDS-9 | 0.000 | 0.000 | 0.000 | 0.000 | 0.000 | 0.000 | 0.000 | 0.404 | 0.106 |  | 0.000 | 0.000 | 0.000 | 0.000 | 0.091 | 0.000 | 0.000 |
| QIDS-10 | 0.000 | 0.032 | 0.000 | 0.057 | 0.029 | 0.085 | 0.000 | 0.030 | 0.099 | 0.000 |  | 0.171 | 0.000 | 0.111 | 0.186 | 0.154 | 0.143 |
| QIDS-11 | 0.000 | 0.040 | 0.000 | 0.000 | 0.000 | 0.283 | 0.000 | 0.086 | 0.000 | 0.000 | 0.171 |  | 0.168 | 0.081 | 0.000 | 0.000 | 0.102 |
| QIDS-12 | 0.045 | 0.000 | 0.080 | 0.000 | 0.105 | 0.258 | 0.000 | 0.000 | 0.000 | 0.000 | 0.000 | 0.168 |  | 0.071 | 0.049 | 0.000 | 0.000 |
| QIDS-12 | 0.088 | 0.041 | 0.000 | 0.000 | 0.061 | 0.106 | 0.089 | 0.045 | 0.000 | 0.000 | 0.111 | 0.081 | 0.071 |  | 0.270 | 0.194 | 0.000 |
| QIDS-14 | 0.000 | 0.000 | 0.000 | 0.000 | 0.081 | 0.117 | 0.000 | 0.089 | 0.000 | 0.091 | 0.186 | 0.000 | 0.049 | 0.270 |  | 0.229 | 0.000 |
| QIDS-15 | 0.000 | 0.000 | 0.000 | 0.065 | 0.000 | 0.035 | 0.076 | 0.000 | 0.000 | 0.000 | 0.154 | 0.000 | 0.000 | 0.194 | 0.229 |  | 0.000 |
| QIDS-16 | 0.000 | 0.073 | 0.000 | 0.000 | 0.000 | 0.000 | 0.000 | 0.000 | 0.000 | 0.000 | 0.143 | 0.102 | 0.000 | 0.000 | 0.000 | 0.000 | 0 |

Network Weights (Four Weeks)
